# PIEZO1 mechanical insensitivity in generalized lymphatic dysplasia with the potential for pharmacological rescue

**DOI:** 10.1016/j.isci.2025.113110

**Published:** 2025-07-15

**Authors:** Melanie J. Ludlow, Oleksandr V. Povstyan, Deborah M. Linley, Silvia Martin-Almedina, Charlotte Revill, Kevin Cuthbertson, Katie A. Smith, Emily Fay, Elisavet Fotiou, Andrew Bush, Claire Hogg, Tobias Linden, Natalie B. Tan, Susan M. White, Juan C. Del Rey Jimenez, Ege Sackey, Esther Dempsey, Sahar Mansour, Gregory Parsonage, Antreas C. Kalli, Richard Foster, Pia Ostergaard, David J. Beech

**Affiliations:** 1 Leeds Institute of Cardiovascular and Metabolic Medicine, School of Medicine, University of Leeds, Leeds LS2 9JT, UK; 2 School of Health & Medical Sciences, City St George’s, University of London, London SW17 0RE, UK; 3 School of Chemistry, University of Leeds, Leeds LS2 9JT, UK; 4 Paediatric Respiratory Medicine, NHLI, Imperial College London, London, UK; 5 Department of Paediatric Respiratory Medicine, Royal Brompton Hospital, London SW3 6NP, UK; 6 Department of Neuropediatrics, University Children’s Hospital, Klinikum Oldenburg, Oldenburg, Germany; 7 Victorian Clinical Genetics Services, Murdoch Children’s Research Institute, Parkville, VIC, Australia; 8 South West Thames Regional Centre for Genomics, St George’s University Hospitals NHS Foundation Trust, London SW17 0QT, UK

**Keywords:** Pharmacology, Cell biology

## Abstract

*PIEZO1* variants have been associated with generalized lymphatic dysplasia (GLD) through mechanisms involving reduced PIEZO1 expression. Here, we report variants where the mechanism involves reduced channel mechanical sensitivity. Two of the variants encode amino acid changes in the channel’s cap structure (Ile2270Thr and Arg2335Gln), one in the ninth transmembrane helical unit (THU) below the cap (Gly1978Asp) and one in the fifth THU distant from the cap (Glu829Val). Patch-clamp studies of the cap and sub-cap variant channels revealed abolished or reduced channel mechanical sensitivity with the possibility to activate the channels and partly rescue mechanical sensitivity by the small molecule Yoda1. The potency of Yoda1 at the variant channels was less than at the wild-type channel, but chemical synthesis of Yoda1 analogs revealed a molecule with improved potency. The data suggest cases of GLD in which there is decreased channel mechanical sensitivity and the potential to reduce dysfunction pharmacologically.

## Highlights

• *PIEZO1* variants associated with GLD are identified, and their pathogenicity is confirmed
• Three of the variants encode changes that reduce PIEZO1 activation by mechanical force
• PIEZO1 agonist Yoda1 activates and partly rescues the mechanical activity of variant channels
• Chemical modification of Yoda1 improves the activation of variant channels

## Graphical abstract

**Figure:**
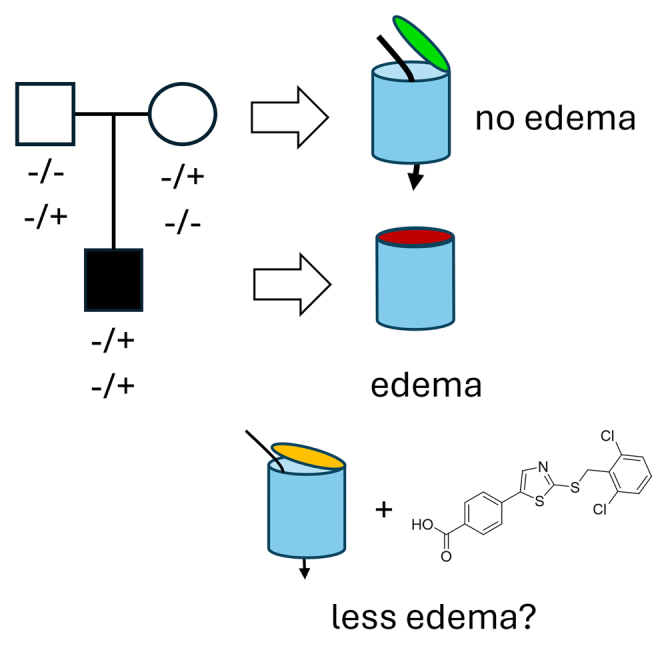

## Introduction

Lymphatics mediate fluid homeostasis, dietary fat absorption, reverse cholesterol transport, and immune cell surveillance and trafficking.^1^^,^^2^^,^^3^^,^^4^ They begin with blind-ended capillaries made of endothelial cells that converge into peristaltic collecting vessels that pass contents through lymph nodes, ultimately to the great veins of the neck.^1^^,^^5^ This system is efficient, but there are estimated to be about 250,000 individuals with chronic edema in the UK^6^ and potentially about 230 million sufferers worldwide.^7^ The causes are multiple, but they include genetic variations such as those of generalized lymphatic dysplasia (GLD) and disruptions to lymphatics caused by factors such as obesity, inflammation, fibrosis, and physical injury.^1^^,^^8^^,^^9^ There is often reduced quality of life and sometimes life-threatening cellulitis and psychological morbidity.^1^ Current therapies focus on the management of symptoms through diet, the wearing of compression garments, massage, skin care, and exercise.^10^ Unfortunately, limited knowledge of the underlying molecular mechanisms of lymphedema has hindered the development of potential lymphatic-targeted medicines that could stimulate lymphatics and reduce unwanted fluid retention, potentially averting detrimental accumulations of fat and immune cells in tissues.

Opportunities for the understanding of lymphedema and devising treatment strategies for it are arising through genetic analysis of GLD where pathogenic variants in at least 16 genes are associated with the disease.^1^ One of the most frequently affected genes is *PIEZO1*.^1^^,^^11^^,^^12^^,^^13^ It encodes the PIEZO1 protein, which is a large membrane protein that assembles as trimers to form mechanically activated calcium ion (Ca^2+^)-permeable non-selective cationic channels.^14^ The channels have remarkable structural features that include the membrane-embedded blades that confer the ability to sense and respond to mechanical forces and a cap (C-terminal extracellular domain) over the top of the ion pore region that is coupled via foot-like structures to the blades.^14^^,^^15^^,^^16^ PIEZO1 is widely expressed,^14^ but results of human and mouse genetic studies suggest that a major consequence of PIEZO1 deficiency is the disruption of lymphatics or lymphatic functions.^11^^,^^12^^,^^17^ Reasons for the lymphatic vulnerability may be the exceptional dependencies of lymphatic permeability and valve mechanisms on PIEZO1’s sensing of subtle forces arising from lymph flow and pressure.^17^^,^^18^^,^^19^ It nevertheless remains unclear if PIEZO1’s force-sensing ability per se is what specifically mediates its special roles in lymphatics.^19^ Moreover, while *PIEZO1* variants have been associated with GLD,^13^ there is limited information about the consequences of these variants for *PIEZO1* expression or PIEZO1 channel function. In some instances, the implications of the variants are obvious such as when the altered gene sequence leads to a premature termination codon,^11^ but in others, there is a lack of clarity, and the variant may only be classified as a variant of uncertain significance (VUS) or likely pathogenic (LP),^20^ thus impeding a molecular diagnosis.

For a small number of GLD families, there are laboratory results showing that variants may act by disrupting the formation of PIEZO1 protein or hindering its trafficking to or stability at the key site of activity, the cell surface membrane (i.e., the plasma membrane).^11^^,^^12^^,^^18^ These mechanisms of disruption are established, but they contrast with the PIEZO1 gain-of-function mechanism of dehydrated hereditary stomatocytosis (DHS) in which adverse effects on red blood cells arise from changes in PIEZO1 channel activity such as the slowing of the channel’s inactivation, which reduces the desensitization of the channels in response to sustained force.^21^^,^^22^ Modified channel activity is also a common mechanism of other ion channelopathies.^23^ We therefore hypothesized that there might also be modified PIEZO1 ion channel activity mediating GLD.

Through further clinical investigation of GLD-affected families and sequencing of their *PIEZO1* exons, we identified associations between GLD and four previously unstudied *PIEZO1* missense variants. Because the variants were classified as VUS or LP, we performed western blotting, intracellular Ca^2+^ measurements, and patch-clamp electrophysiology to investigate their effects experimentally. Based on the results, we suggest modified ion channel activity in GLD and, more specifically, a modified cap behavior in which variants act by altering the PIEZO1 cap or its association with the force-sensing blades. Importantly, these mechanically insensitive channels could still be activated pharmacologically by the Yoda1 small molecule, which is a synthetic chemical identified from screening 3.25 million compounds.^24^ Yoda1 is particularly potent at activating PIEZO1 in lymphatic endothelial cells,^25^ and this supports the idea of it potentially selectively stimulating lymphatics despite the broad expression of PIEZO1,^14^ and it has been shown to reduce peripheral and central edemas in mice.^18^^,^^26^^,^^27^ Analogs of Yoda1 have been generated to explore preliminary structure-activity relationships of PIEZO1 agonists and develop modulators of PIEZO1 channels that are physicochemically or pharmacologically improved, or simply different.^28^^,^^29^^,^^30^^,^^31^ Research of this type informs understanding of the chemical requirements for channel modulation, and it potentially enables the discovery of therapeutic drug-like molecules that could enter clinical trials for lymphedema.^31^

## Results

### *PIEZO1* variants associated with GLD

**Figure 1:**
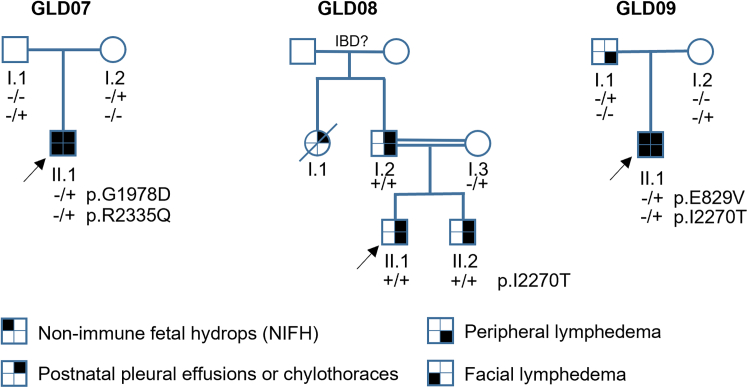
Pedigrees of GLD families GLD07–09 Affected individuals are indicated with filled circles or squares. *PIEZO1* genotypes are indicated for individuals who underwent Sanger sequencing. The wild-type allele of the genotype is indicated by minus sign (−), and plus (+) represents the alternative allele. Arrows indicate the proband. IBD indicates the unconfirmed identity by descent.

Since our initial description of *PIEZO1* variants in 6 GLD families (GLD01–06),^11^ more families came to our attention. Here, we report on 3 more families (GLD07–09) (Figure 1; Table 1 and further clinical information in the STAR Methods). DNA sequencing identified *PIEZO1* missense variants associated with the disease (Figure 1; Table 1, STAR Methods; Figure S1). In GLD07, compound heterozygosity of variants encoding Gly1978Asp (G1978D) and Arg2335Gln (R2335Q) associated with disease in one family member (Figure 1). In GLD08, homozygosity of a single variant encoding Ile2270Thr (I2270T) associated with disease in 3 family members (Figure 1). In GLD09, heterozygosity of a single variant encoding Glu829Val (E829V) or compound heterozygosity of this variant with I2270T associated with disease in 2 family members (Figure 1). The variants were interpreted using best practice guidelines^20^ as LP or VUS (Table S1). There were diverse disease features of the families (Figure 1; Table 1, Figure S1 and further clinical information in the STAR Methods). In GLD07 and GLD09, there was non-immune fetal hydrops (NIFH) and facial lymphedema, whereas in GLD08, there was disease only from adolescence that was characterized first by pleural and pericardial effusions and then lower limb and scrotal edema without facial edema. The data suggested disease-causing variants that were not sufficiently understood nor proven to have a pathogenic impact. We therefore focused on laboratory investigation of the variant effects, first studying I2270T because this variant was previously reported in *trans* with a frameshift variant in an NIFH case,^32^ and because of its segregation with disease in GLD08 and GLD09, particularly the singular association with disease in GLD08.

**Table 1:** Clinical and genetic findings in patients with GLD with *PIEZO1* missense variants

Family	ID	Sex	Current age	Genotyping		Antenatal history		Neonatal history	Lymphedema (postnatal and onward)					Additional clinical features	
				Variant	Zygosity	NIFH	PH	Peripheral edema	Age of onset	Limbs	Face	Scrotal/genital	PE/CT	Dys-morphic features	Other comments
GLD07	II.1	M	13 y	c.5933G>A p.(G1978D); c.7004G>A p.(R2335Q)	Comp- Het	Y	Y	Y	<1 y	bilateral lower limbs, mainly feet	Y	intermittent	Y	N	maldescended testis left side, perihepatic ascites
GLD08	I.2	M	55 y	c.6809T>C; p.(I2270T)	Hom	N	?	N	30 y	bilateral lower limbs	N	intermittent	Y	N	mild pericardial effusions, varicose veins with eczema
GLD08	II.1	M	30 y	c.6809T>C; p.(I2270T)	Hom	N	?	N	11 y	bilateral lower limbs	N	scrotal edema	Y	N	ascites, pericardial effusion leading to cardiac tamponade
GLD08	II.2	M	17 y	c.6809T>C; p.(I2270T)	Hom	N	N	N	12 y	bilateral lower limbs	N	hydrocoeles	Y	Y	bilateral chylothoraces
GLD09	I.1	M	39 y	c.2486A>T; p.(E829V)	Het	N	N	Y	birth	four limbs	N	N	N	N	upper limb swelling in adulthood
GLD09	II.1	M	7 y	c.2486A>T; p.(E829V); c.6809T>C; p.(I2270T)	Comp- Het	Y; bilateral hydrothoraces (19 wk) and facial edema	Y	Y	birth	bilateral lower limbs	intermittent	Y	Y	N	severe GOR requiring fundoplication and gastrostomy. AS, metopic, craniosynostosis, OSA

Variants are annotated to the NM_001142864.4 reference transcript and displayed as nucleotide and amino acid changes.

Comp-Het, compound heterozygous; Hom, homozygous; Het, heterozygous; NIFH, non-immune fetal hydrops; PH, polyhydramnios; PE/CT, pleural effusion/chylothoraces; GOR, gastroesophageal reflux; AS, Asperger syndrome; OSA, obstructive sleep apnoea; M, male; wk, week; y, year; Y, yes; N, no; ?, information not available.

### I2270T localizes to the PIEZO1 cap

There are partial structural data for human PIEZO1 (hPIEZO1)^33^ and mouse PIEZO1 (mPIEZO1) channels,^14^^,^^15^^,^^34^^,^^35^^,^^36^ which are similar.^33^ We used these data to inform the construction of a model of the hPIEZO1 channel (Figure S2). The model localizes I2270T to the channel’s cap (Figure S2). Previous clinical investigation identified a homozygous missense variant (M2225R) in the cap that associated with DHS, and amplified channel function,^21^^,^^22^^,^^37^ which is opposite to the effect expected in GLD.^11^^,^^12^ The data suggest the localization of I2270T to a key structural domain of the channel (the cap) but continued uncertainty about whether this amino acid change would disrupt the channel.

### Loss of mechanical activation

**Figure 2:**
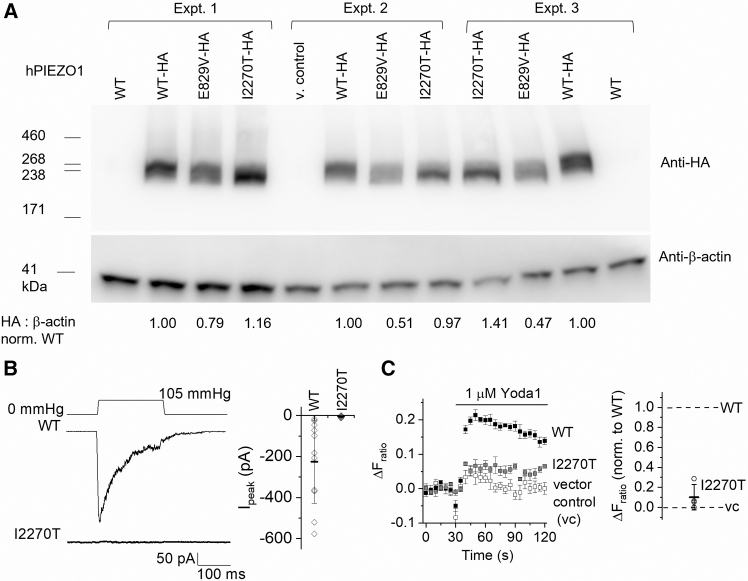
Expression but mechanical resistance of I2270T hPIEZO1 (A) Western blot data for untagged and hemagglutinin (HA)-tagged wild-type (WT) hPIEZO1 and its E829V and I2270T variants transiently expressed in modified HEK293 (T-REx-293) cells and immunoblotted with anti-HA antibody (upper) and anti-β-actin antibody (lower, loading control). Data are shown for 3 independent experiments (experiments 1, 2, 3). The numbers below the lower gel are for the anti-HA band intensity divided by the β-actin band intensity normalized to WT-HA (mean ± SD values for E829V-HA and I2270T-HA are 0.59 ± 0.17 and 1.18 ± 0.22, respectively). (B) Left: example ionic currents in outside-out patch recordings from T-REx-293 cells transiently expressing WT or I2270T hPIEZO1 exposed to the 105-mmHg pressure pulse shown schematically at the top. Right: summary data for the types of experiment shown on the left. Amplitude of the peak current is represented as mean ± SD and each independent data point is superimposed (WT *n* = 11, I2270T *n* = 4). (C) Left: increase in intracellular Ca^2+^ concentration indicated by increase in the fura-2 fluorescence (F) ratio above baseline (ΔF_ratio_) in T-REx-293 cells transiently transfected with WT hPIEZO1, I2270T hPIEZO1, or empty vector (vector control). Cells were stimulated with 1 μM Yoda1. Example data are shown for a single representative 96-well plate experiment (mean ± SEM, *N* = 4–5 wells each). Right: summary data for experiments of the type shown on the left for the signal measured between 30 and 60 s after Yoda1 application (*n* = 4 independent experiments). Data are mean ± SD normalized to the respective WT channel data and subtracted for the amplitude in the vector control (vc) group.

To test for effects of I2270T on PIEZO1, we mutated hPIEZO1 cDNA in a plasmid and overexpressed it in modified HEK293 (T-REx-293) cells, incorporating an integrated hemagglutinin (HA) tag to unambiguously identify the protein in western blotting using an anti-HA antibody. The abundance of I2270T hPIEZO1 was at least that of non-variant wild-type (WT) hPIEZO1, suggesting that expression was not adversely affected by the mutation (Figure 2A). By contrast, the E829V variant that is compound heterozygous with I2270T in the GLD09 proband and associated with disease in the proband’s father (Figure 1) showed reduced expression (Figure 2A). To investigate if I2270T affects channel activity instead, ionic currents were recorded from excised outside-out membrane patches to which positive pressure pulses were applied, which stretched the membrane and thereby applied force.^38^^,^^39^ WT hPIEZO1 channels were activated by the pulses and showed the expected rapid activation (i.e., an initial fast increase in inward current) followed by inactivation (i.e., a progressive loss of inward current despite sustained pressure) (Figure 2B). By contrast, I2270T channels showed no response (Figure 2B). The data suggest that I2270T abolishes mechanical activation of the channels without affecting protein expression.

### Activation by Yoda1

I2270T’s location does not overlap with the predicted interaction site of the PIEZO1 small-molecule agonist Yoda1 (Figure S2),^40^ so we investigated the activation of I2270T channels by Yoda1. Intracellular Ca^2+^ was recorded from hPIEZO1-overexpressing or vector control (non-expressing) cells, and Yoda1 was tested at 1 μM, which is just above the threshold for activation of WT channels,^28^^,^^29^ thereby maximizing the sensitivity of the assay to changes in channel function. I2270T channels responded poorly compared with WT channels but just above the background of the vector control cells, suggesting the potential of Yoda1 to restore at least some of the variant channel’s function (Figure 2C). To investigate further, we turned to mPIEZO1 as a surrogate because it is more extensively characterized,^14^ similar in structure to hPIEZO1^33^ and previously used to aid the study of human variants.^41^ The I2270 residue and adjacent amino acid residues in the PIEZO1 sequence are evolutionarily conserved across diverse species (Figure S3), supporting the use of mPIEZO1. Most importantly for our studies, mPIEZO1 exhibits better sensitivity to Yoda1 than hPIEZO1,^29^ thus enabling the construction of full concentration-response curves despite Yoda1’s ∼2 μM aqueous solubility limit, and thereby the quantification of agonist sensitivity as the concentration of agonist causing 50% effect (EC_50_).^29^^,^^30^^,^^31^

**Figure 3:**
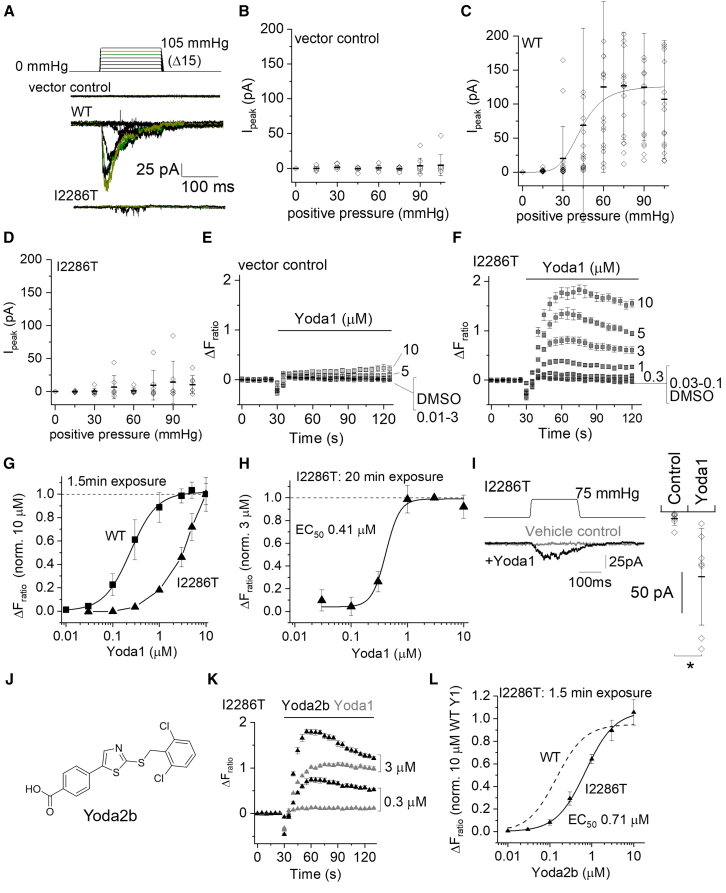
Mechanical resistance but pharmacological activation of I2286T mPIEZO1 (A–D and I) Data for outside-out patch recordings from T-REx-293 cells stably transfected with empty vector (vector control), wild-type (WT) mPIEZO1, or I2286T mPIEZO1. The voltage across each membrane patch was −80 mV. (A) Upper: pressure pulse protocol in which a 200-ms pulse was applied to 15 mmHg and then incremented (Δ) every 12 s in steps of 15 mmHg up to a maximum of 105 mmHg. Lower: example ionic currents from patches excised from cells transfected with empty vector (vector control), WT mPIEZO1, or I2286T mPIEZO1. Currents evoked by 75 and 90 mmHg are colored in green and olive, respectively. (B–D) For experiments of the type exemplified in (A), peak current amplitudes plotted against pressure and shown as mean ± SD with individual data points for each experiment superimposed (*n* = 9 for vector control, *n* = 15–17 for WT, and *n* = 7 for I2286T). The smooth curve in (C) is a fitted Boltzmann function with mid-point (P_50_) at 42.4 mmHg. (E and F) Example data for the increase in intracellular Ca^2+^ concentration indicated by increase in the fura-2 fluorescence (F) ratio above baseline (ΔF_ratio_) in T-REx-293 cells stably transfected with empty vector (vector control) (E) or I2286T mPIEZO1 (F). Mean ± SEM and *N* = 4–5 wells each. (G) Summary mean ± SEM concentration-response data for experiments of the type shown in (F) with a Hill equation fitted to the WT mPIEZO1 data (EC_50_ 0.24 μM) and data points for I2286T mPIEZO1 joined by straight lines (*n* = 3–6). (H) As for (G) but I2286T mPIEZO1 only and for long (20 min) exposure to Yoda1. Data are normalized to the response to 3 μM Yoda1. The curve is a fitted Hill equation, generating an EC_50_ of 0.41 μM (*n* = 5–6). (I) Left: data for a patch exposed to 75 mmHg pressure pulses in the presence of DMSO (vehicle control) and then 5 μM Yoda1 (+Yoda1). Right: for experiments of the type exemplified on the left, the maximum amplitude of current evoked by the pressure pulse. Mean ± SD with each independent data point superimposed (*n* = 7). ∗*p* < 0.05 (paired Student’s t test). (J) Chemical structure of Yoda2b (CHR-1871-032). (K) Similar to the approach of (F) but side-by-side comparison of the effects of Yoda2b and Yoda1 on I2286T mPIEZO1 in the same 96-well plate (mean ± SEM, *N* = 4–5 wells each). (L) Similar to (G) except using Yoda2b instead of Yoda1 and for I2286T mPIEZO1 only (*n* = 3). The fitted Hill equation yielded an EC_50_ of 0.71 μM. The dashed curve is the fitted Hill equation from Figure S6B for WT mPIEZO1 (EC_50_ 0.14 μM).

The murine equivalent of I2270T (I2286T) was generated in mPIEZO1 and also overexpressed in the same modified HEK293 cells. It similarly prevented mechanical activation of these channels (Figures 3A–3D). These channels were robustly activated by Yoda1 (Figures 3E–3G). The concentration-response curve was, however, to the right of that for the WT channels (Figure 3G). The WT channels were activated maximally within the aqueous solubility limit of Yoda1, but we did not observe a saturating maximum effect for the variant channel even at 10 μM Yoda1, preventing determination of the EC_50_ and thus quantification of potency (Figure 3G). In an attempt to overcome this limitation, we increased the equilibration time with Yoda1 from 1.5 to 20 min, reasoning that it may allow more complete access of the Yoda1 via a putative lipid barrier to its presumed interaction site on PIEZO1 (Figure S2).^31^ A saturating concentration-response curve (i.e., one that reached a maximum effect) was now observed within the solubility limit of Yoda1, and the EC_50_ was determined to be 0.41 μM (Figure 3H). The data are consistent with this variant PIEZO1 being expressed and available for pharmacological activation.

### Mechanical activation in the presence of Yoda1

PIEZO1 channels serve mechanical force-sensing roles in cells,^14^ and the actions of Yoda1 and mechanical force synergize.^24^^,^^31^ Therefore, we tested whether Yoda1 recovers the mechanical sensitivity of the I2286T channels. While Yoda1 failed to activate the channels in outside-out patches (i.e., there was no change in baseline current), pressure-evoked current now occurred (Figure 3I). The data suggest that Yoda1 at least partly rescues mechanical force sensitivity in I2286T channels.

### Improved rescue by Yoda2b

I2286T channel properties were not fully returned to those of WT channels (Figures 3G and 3I) and so we sought improvement. Analogs of Yoda1 have previously been generated that are more efficacious and potent than Yoda1 on WT PIEZO1 channels.^29^^,^^30^^,^^31^ Based on prior knowledge of the chemical structure-activity relationships of such analogs,^28^^,^^29^^,^^31^ seven analogs of Yoda1 were designed (Figure 3J; Figure S4) and synthesized as described in the STAR Methods. For analog KC124, the chlorine atoms of the righthand aromatic ring were conservatively modified, while in the other analogs, the lefthand ring was more extensively modified including incorporation of various substituted aryl and arylcarboxamide groups, where greater variation is possible without loss of efficacy.^31^ Yoda2b incorporated a modified central ring with 1,3-thiazole (Figure 3J) replacing the 1,3,4-thiadiazole of Yoda1 (Figure S4). The analogs were first tested at 10 μM alongside Yoda1 and Dooku1^28^ on the WT channels with 20-min exposure to the substance in a Ca^2+^ assay and in a hypotonic assay designed to mimic edema (Figures S5 and S6). One of the analogs, Yoda2b (Figure 3J), was about 25% more effective than Yoda1 under the hypotonic condition (Figure S6) and so it was selected for further investigation. We refer to it as Yoda2b because of its chemical similarity to the previously reported Yoda2.^29^^,^^31^ In a side-by-side comparison, Yoda2b was more effective at activating I2286T channels than Yoda1 (Figure 3K). Even with short duration exposure in physiological tonic salt solution, a complete concentration-response curve was now possible, yielding an EC_50_ of 0.71 μM, and there was closer alignment to the equivalent WT data (Figure 3L compared with Figure 3G). Chemical modifications also have the potential to improve physicochemical properties.^29^ Yoda2b had a kinetic solubility at physiological pH in PBS of 6.1 μM and a mouse microsomal stability half-life of 7.2 min. It was 0.4% unbound to mouse plasma proteins. These are all improvements over Yoda1.^29^ The data suggest the potential to improve the rescue capability of Yoda1 through medicinal chemistry strategies.

### Similar effect of cap-localized R2335Q

**Figure 4:**
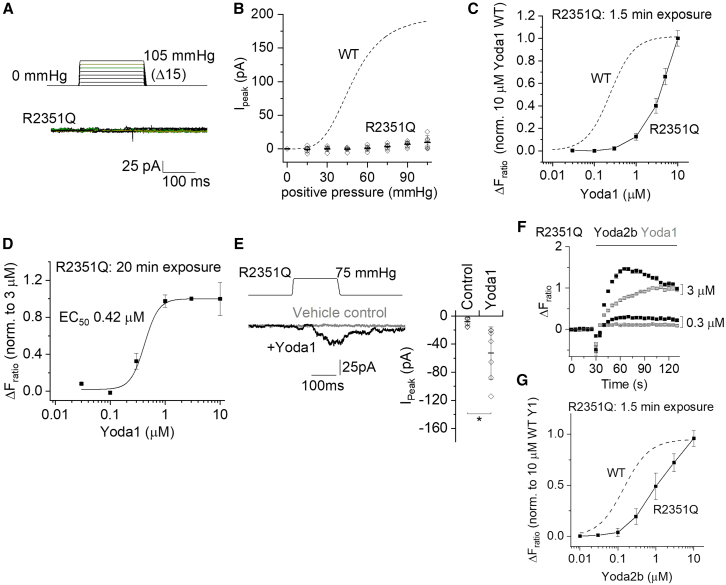
Mechanical resistance but pharmacological activation of R2351Q mPIEZO1 (A and B) Data for outside-out patch recordings from T-REx-293 cells stably transfected with R2351Q mPIEZO1. The voltage across each membrane patch was −80 mV. (A) Upper: 200-ms pressure pulse protocol applied to 15 mmHg and then incremented (Δ) every 12 s in steps of 15 mmHg up to a maximum of 105 mmHg. Lower: example ionic currents from a patch excised from a cell transfected with R2351Q mPIEZO1. Currents evoked by 75 and 90 mmHg are colored in green and olive, respectively. (B) For experiments of the type shown in (A), quantification of peak current amplitude plotted against pressure and shown as mean ± SD with individual data points for each experiment superimposed (*n* = 6 for R2351Q). The dashed curve is the fitted Hill equation from Figure 3C for WT mPIEZO1. (C, D, F, and G) Data for the increase in intracellular Ca^2+^ concentration indicated by the increase in the fura-2 fluorescence (F) ratio above baseline (ΔF_ratio_) in T-REx-293 cells stably transfected with R2351Q. (C) Summary concentration-response data with data points joined by straight lines. Mean ± SEM and *N* = 4–5 wells each (*n* = 3–6). The dashed curve is the fitted Hill equation from Figure 3G for WT mPIEZO1. (D) As for (C) but with long (20 min) exposure to Yoda1. Data are normalized to the response to 3 μM Yoda1. The curve is a fitted Hill equation, generating an EC_50_ of 0.42 μM (*n* = 5–6). (E) Left: data for a patch exposed to 75 mmHg pressure pulses in the presence of DMSO (vehicle control) and then 5 μM Yoda1. Right: for experiments of the type shown on the left, maximum amplitude of current evoked by the pressure pulse. Mean ± SD with each independent data point superimposed (*n* = 7). ∗*p* < 0.05 (paired Student’s t test). (F) Side-by-side comparison of the effects of Yoda2b and Yoda1 on R2351Q mPIEZO1 on the same 96-well plate (mean ± SEM, *N* = 4–5 wells each). (G) Similar to (C) except using Yoda2b instead of Yoda1 (*n* = 3). The dashed curve is the fitted Hill equation from Figure S6B for WT mPIEZO1.

We next considered R2335Q of the GLD07 family (Figure 1) because it is also localized to the cap structure (Figure S2). Similarly, its recapitulation in mPIEZO1 (R2351Q) abolished mechanical activation of the channel (Figures 4A and 4B). Short-duration 1.5-min exposure to Yoda1 caused Ca^2+^ elevation in cells overexpressing this variant, but the concentration-response curve was to the right of the WT curve and the curve did not saturate even at 10 μM Yoda1 (Figure 4C). Similar to I2286T, long-duration 20-min exposure to Yoda1 improved the concentration-response curve, yielding an EC_50_ of 0.42 μM (Figure 4D). Moreover, in the presence of Yoda1, there was robust mechanical activation of the variant channels (Figure 4E). The R2351Q channel was similarly more potently activated by Yoda2b (Figure 4F), although the concentration-response curve did not saturate, which prevented us from determining the EC_50_ (Figure 4G). The data suggest that R2335Q acts similarly to I2270T, preventing mechanical activation while enabling partial rescue by Yoda1 and improved rescue by Yoda2b.

### Lesser effect of sub-cap G1978D

**Figure 5:**
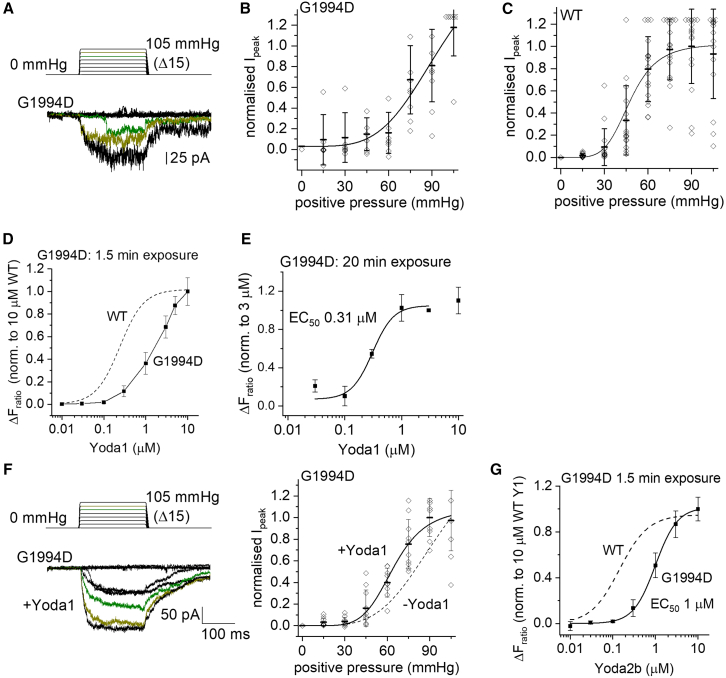
Reduced mechanical sensitivity and pharmacological activation of G1994D mPIEZO1 (A–C) Data for outside-out patch recordings from T-REx-293 cells stably transfected with G1994D mPIEZO1 (A and B) or WT mPIEZO1 (C). The voltage across each membrane patch was −80 mV. (A) Upper: 200-ms pressure pulse protocol applied to 15 mmHg and then incremented (Δ) every 12 s in steps of 15 mmHg up to a maximum of 105 mmHg. Lower: example ionic currents from a patch excised from a cell transfected with G1994D mPIEZO1. Currents evoked by 75 and 90 mmHg are colored in green and olive, respectively. (B and C) For experiments of the type shown in (A), quantification of peak current amplitude plotted against pressure for G1994D mPIEZO1 (B) and WT mPIEZO1 (C) normalized to the maximum I_peak_ value and shown as mean ± SD with individual data points for each experiment superimposed (*n* = 7–8 for G1994D, *n* = 15–17 for WT). A single Boltzmann function is fitted to the G1994D data, but no P_50_ is indicated because current saturation did not occur. The fitted Boltzmann function to the WT data had a mid-point (P_50_) at 49.4 mmHg. (D, E, and G) Data for the increase in intracellular Ca^2+^ concentration indicated by increase in the fura-2 fluorescence (F) ratio above baseline (ΔF_ratio_) in T-REx-293 cells stably transfected with G1994D mPIEZO1). (D) Summary concentration-response with data points joined by straight lines. Mean ± SEM and *N* = 4–5 wells each (*n* = 3–6). The dashed curve is the fitted Hill equation from Figure 3G for WT mPIEZO1. (E) As for (D) but using long (20 min) exposure to Yoda1. Data are normalized to the response to 3 μM Yoda1. The curve is a fitted Hill equation, generating an EC_50_ of 0.31 μM (*n* = 5–6). (F) Left: data for a patch exposed to incrementing pressure pulses from 15 to 105 mmHg pressure pulses in the presence of 5 μM Yoda1. Right: for experiments of the type shown on the left, mean and individual data of the type in (B) but in 5 μM Yoda1. The smooth curve is a fitted Boltzmann function with mid-point (P_50_) of 64.0 mmHg. The dashed curve is the Boltzmann fit to data for G1994D without Yoda1 (−Yoda1) from (B). Mean ± SD with each independent data point superimposed (*n* = 9–10). (G) Similar to (D) except using Yoda2b (*n* = 3). The dashed curve is the fitted Hill equation from Figure S6B for WT mPIEZO1.

G1978D, which alongside R2335Q associates with disease in GLD07 (Figure 1), localizes to the membrane-spanning THU9 below the cap (Figure S2). Its recapitulation in mPIEZO1 (G1994D) reduced but did not abolish the mechanical sensitivity of the channels (Figure 5A), right-shifting the pressure-response curve (Figure 5B) relative to that of the WT channels (Figure 5C). It simultaneously reduced the inactivation property of the channels during a sustained pressure step (Figure 5A), contrasting with the striking inactivation often seen in WT channels (Figure 3A). Loss of inactivation is a gain-of-function effect.^21^ The data suggest that more pressure is required to activate the G1994D channel but that the channels then remain open for longer. Therefore, there is a mixed loss- and gain-of-function effect of this mutation, suggesting a less severe overall effect than mutations I2270T and R2335Q.

Short-duration 1.5-min exposure to Yoda1 elevated Ca^2+^ in cells expressing the G1994D channel, but the concentration-response curve was right-shifted compared with WT, and saturation of the effect did not occur even at 10 μM Yoda1 (Figure 5D). Long-duration 20-min exposure to Yoda1 improved the concentration-response curve, and the EC_50_ was determined as 0.31 μM (Figure 5E). Yoda1 left-shifted the pressure-response curve (Figure 5F), making the pressure for 50% activation (P_50_) measurable at 64.0 mmHg, although there was still less sensitivity than WT channels (P_50_ 49.4 mmHg, Figure 5C). A complete concentration-response curve for Yoda2b was achieved with short exposure, yielding an EC_50_ of 1.0 μM (Figure 5G). The data suggest that G1978D has two opposing effects on the electrophysiological properties of the channels and that it reduces but does not prevent activation by Yoda1 and Yoda2b.

## Discussion

**Figure 6:**
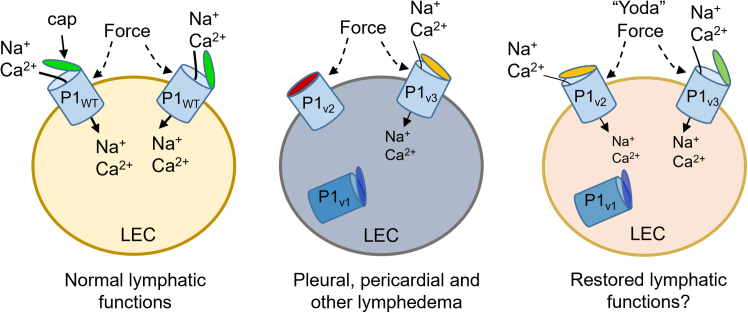
Model for *PIEZO1* variant effects and the potential for a therapeutic strategy targeted to PIEZO1 Left: physiological lymphatic endothelial cell (LEC) with wild-type sodium and calcium (Na^+^ and Ca^2+^)-permeable PIEZO1 channels (P1_WT_) that have active caps that are important for channel activation by mechanical force. The ion fluxes through the channels stimulate physiological activities of the LECs. Middle: lymphedema LEC containing PIEZO1 variants of 3 types: P1_v1_ (reduced expression, e.g., E829V), P1_v2_ (disruption to the cap, strongly reducing mechanical activation—I2270T or R2335Q), and P1_v3_ (disruption to the cap-associated blade, partially reducing mechanical sensitivity—G1978D). Because of these defects, there is less Na^+^ and Ca^2+^ entry and thus reduced physiological activities of the LECs, leading to lymphatic dysfunctions that are seen as pleural and pericardial effusions and other features of GLD. Right: improvement in P1_v2_ and P1_v3_ functions due to the presence of a Yoda small-molecule agonist (e.g., Yoda2b) that stimulates these channels and has the potential to at least partially restore physiological LEC and lymphatic activities. Left, middle, and right: a functional cap is indicated by green color and a partially functional cap by orange and a loss of functional cap by red. Deeper blue indicates a trapped or disrupted channel in intracellular compartments.

This study reveals GLD-affected families in which disease associates with *PIEZO1* missense variants that adversely affect PIEZO1 channel activity. The findings add to prior knowledge of the clinical and mechanistic effects of *PIEZO1* variants in GLD and support the idea for a potential therapy that might have implications in and beyond these rare cases of GLD. In Figure 6, we suggest a model for *PIEZO1* variant-related GLD based on our results presented here (summarized in Table 2) and the results of other published studies.^11^^,^^12^^,^^17^^,^^18^^,^^25^^,^^26^^,^^42^ In the model of Figure 6, *PIEZO1* is expressed in lymphatic endothelial cells, leading to PIEZO1 channels at the surface membrane that confer sensitivity to mechanical forces (e.g., from lymphatic pressure and flow) and transduction of the amplitudes of these forces into effects via cation fluxes and downstream cellular events that ensure suitable lymphatic functions.^1^^,^^17^^,^^19^^,^^25^ While some *PIEZO1* variants disrupt *PIEZO1* expression or otherwise inhibit PIEZO1’s formation or its localization to the cell surface,^11^^,^^12^ we suggest further molecular etiology of GLD in which PIEZO1 is expressed and reaches the surface membrane but is then unable, or less able, to sense mechanical force. The GLD associated with these different types of *PIEZO1* disruption varies in its characteristics, potentially in part because of the range of ways in which PIEZO1 is affected, from apparently severe depletion or complete absence of PIEZO1, to its presence but with reduced capability. In the latter, when channels are present but less capable, we suggest an opportunity for intervention using PIEZO1 small-molecule agonists.

**Table 2:** Summary of laboratory evidence and PS3 criterion assessment

hPIEZO1 amino acid change	Affected PIEZO1 channel region	Effect on hPIEZO1 channel function	Effect on hPIEZO1 expression	Equivalent mPIEZO1 amino acid change	Effect on mPIEZO1 channel function	Y2b EC_50_ mPIEZO1	PS3 functional evidence score
I2270T	cap	loss of MA: LOF; reduced 1 μM Y1 effect	no effect	I2286T	loss of MA: LOF; reduced Y1 potency	0.71 μM (WT = 0.14 μM)	PS3_moderate
R2335Q	cap	reduced 1 μM Y1 effect	not prevented^a^	R2351Q	loss of MA: LOF; reduced Y1 potency	could not be determined	PS3_moderate
G1978D	THU9	reduced 1 μM Y1 effect	not prevented^a^	G1994D	reduced MA: LOF; reduced inactivation: GOF; reduced Y1 potency	1.0 μM	PS3_moderate
E829V	THU5	reduced 1 μM Y1 effect	reduced	E824V	reduced Y1 potency	0.76 μM	PS3_moderate

Results from the various tests carried out for each variant are summarized here. The strength of the evidence supported a PS3_moderate score for all variants, providing sufficient evidence to classify the VUSs as likely pathogenic (LP) and supporting the pathogenic effect in the other two (Table S1).

hPIEZO1, human PIEZO1; mPIEZO1, mouse PIEZO1; WT, wild type; THU, transmembrane helical unit; MA, mechanical activation; LOF, loss-of-function effect; GOF, gain-of-function effect; Y1, Yoda1; Y2b, Yoda2b; EC_50_, concentration required to cause 50% effect; PS3, a strong criterion used at a moderated level under the ACMG/AMP sequence variant interpretation framework used to evaluate well-established *in vitro* or *in vivo* functional studies that support a damaging effect on the gene or gene product.

a Western blotting was not performed to quantify protein expression; it is rather inferred that expression was not prevented because there were responses to Y2b, which depended on channel expression. See Figure S7 for the “reduced 1 μM Y1 effect” data of R2335Q, G1978D, and E829V hPIEZO1, and E824V, which is the mPIEZO1 equivalent of E829V. See Figure S8 for the Y2b concentration-response data of E824V mPIEZO1.

Most clearly in the case of I2270T, the findings suggest that PIEZO1’s force sensing per se is what mediates its roles in lymphatics, rather than potentially other effects of PIEZO1 that might arise, for example, through PIEZO1 effects on membrane curvature.^43^ Specifically, the GLD08 data suggest that loss of PIEZO1’s mechanical sensitivity due to homozygous I2270T is what causes lymphatic insufficiency in the lungs, heart, and other organs of the affected individuals. Our data suggest that expression of the I2270T variant is similar to that of WT and that the variant is likely to reach the plasma membrane, where it is available for activation by a combination of pressure and Yoda1. Nevertheless, we do not exclude the possibility of the variant also modulating trafficking, which may contribute to the disease if it occurs.

There was no NIFH or other perinatal disease in the GLD08 individuals, contrasting with the NIFH in GLD07 and GLD09 and the suggestion that *PIEZO1* variants are the most common monogenic etiology of NIFH.^13^ Therefore, biallelic *PIEZO1* variants do not always associate with a neonatal onset problem. Similarly, facial edema, another common feature of GLD, was absent in GLD08. Therefore, other unknown properties of PIEZO1 may contribute and have particular significance in some circumstances. It is difficult at this point of time to make a deeper conclusion on genotype and phenotype correlations. Variable phenotypes are common in genetic disease, and we still have too few cases in this large protein, PIEZO1, to make suitable observations. This is why this study, and any future work of a similar kind, is important, as we need to learn more about the different domains of the PIEZO1 protein and what the consequences are of pathogenic variants in the affected regions of the protein. Our study demonstrates that there is considerable complexity, and thus, a lot more cases than what we have available currently are needed for a meaningful discussion on this aspect.

The GLD08 data provide evidence that the PIEZO1 channel is important in human pericardial fluid drainage, consistent with prior observations in mice and zebrafish.^44^^,^^45^ Therefore, the data suggest that there is physiological importance of PIEZO1 in the control of fluid homeostasis around the human heart. Pericardial fluid accumulation^46^ can lead to cardiac tamponade,^47^ which, while rare, may be life-threatening unless there is successful clinical intervention. Targeting of PIEZO1 might present a way to reduce such events. Our data do not directly implicate PIEZO1 in lymphatics of the myocardium, but this may also be worth considering. Cardiac lymphatics are an underinvestigated but important aspect of physiology. An increase in cardiac water content of the heart by only 3% suppresses cardiac output by 30%.^48^ Studies in mice suggest that cardiac lymphatics are pivotal in cardiac recovery after myocardial injury.^49^^,^^50^ In contrast to the pericardial findings, there is prior evidence that PIEZO1 is required for normal pleural fluid homeostasis.^12^^,^^17^

Although we know the cap is important in channel function,^16^ it is somewhat surprising that variants affecting the cap (I2270T and R2335Q) would have such damaging effects on the mechanical activation of the channel. The membrane-embedded blades of the channels are critical in mechanical sensitivity, acting as the force sensors.^14^^,^^15^^,^^36^^,^^51^ The cap sits above the ion pore region and outside the lipid bilayer, but it also projects foot-like structures to the blades, so by this route, the cap could regulate force sensitivity.^14^^,^^15^^,^^52^ One hypothesis is that the cap-localized variants (I2270T and R2335Q) increase the cap association with the blades, thereby reducing blade flexibility and right-shifting the mechanical sensitivity out of the range of physiological forces experienced in lymphatics.

M2225R, a different cap-localized variant that rather associates with DHS, increases channel function by slowing inactivation or deactivation kinetics without affecting the threshold for mechanical activation.^21^^,^^22^^,^^37^^,^^53^ Therefore, cap variants do not necessarily inhibit channel function or regulate mechanical sensitivity. Studies of overexpressed WT and artificial mutant mPIEZO1 and mouse PIEZO2 (mPIEZO2) channels have pointed to structural features and specific amino acid residues that are important in the cap.^16^ Replacement of 3 subdomains at the base of the mPIEZO1 cap by corresponding sequences from mPIEZO2 generated channels with the faster inactivation kinetics of mPIEZO2.^16^ These findings reinforce the idea that the cap is a determinant of the inactivation rate of the channels. The two cap variants studied here (I2286T and R2351Q in mPIEZO1) do not, however, localize to any of these subdomains, nor to other subdomains that were selected based on poor sequence conservation between mPIEZO1 and mPIEZO2.^16^ We can see in our hPIEZO1 and mPIEZO1 model structures that I2270T and I2286T are predicted to be about halfway down the cap and R2335Q and R2351Q at the top in the cap subdomain d^16^ (Figure S2).

Key amino acid residues of mPIEZO1 involved in the interaction between the cap feet and the abutting blade regions are E2257 and D2264 in the cap and R1762 and R1761 in the blade.^15^^,^^16^ Electrostatic interactions between the acidic and basic side chains of these residues are suggested to regulate the mechanical sensitivity of the channels. I2286T and R2351Q, studied here, do not reside at these sites; however, and to the best of our knowledge, I2286T does not reside in any previously investigated region of the channel. I2286T is between subdomains a and b specified in the mPIEZO1/mPIEZO2 chimera study^16^ and 6 residues along from a region specified in another study of mPIEZO1 as the α1-α2 helix of a suggested “cap-gate-loop” (residues 2253–2280).^52^ Combined neutralization of E2279 and D2280 (by mutation to alanine) abolished mechanical sensitivity of the channels,^52^ supporting the idea that the cap is critical for mechanical sensitivity as well as inactivation. Therefore, we hypothesize that I2270T/I2286T may inhibit mechanical sensitivity by somehow disturbing the cap-gate-loop or an associated structure. How an altered cap-gate-loop would inhibit mechanical sensitivity remains to be determined.

In GLD09, I2270T is maternally inherited, and the mother (GLD09 I.2) is unaffected by disease. Therefore, as in GLD08, heterozygosity of I2270T seems not to be associated with disease. The father in GLD09 (GLD09 I.1) is, however, monoallelic for E829V with a history of congenital onset primary lymphedema. While the mechanism of action of E829V was not a focus of this study, the less effective expression seen for E829V PIEZO1 (Figure 2A) may suggest that haploinsufficiency of PIEZO1 is sufficient to cause lymphatic dysfunction in some individuals or that another yet undetected variant exists in the father. E829V reduced the abundance of overexpressed PIEZO1 by about 40% (Figure 2), which is less severe than its reduction of the 1 μM Yoda1 Ca^2+^ response (Figure S7). E829V might therefore have additional mechanisms of action. Studies of the variant recapitulated in mPIEZO1 have suggested that the channel reaches the plasma membrane with a threshold for mechanical activation similar to that of WT channels.^54^ However, the mechanical activation curve was biphasic,^54^ suggesting potential additional alterations to the channel properties that are not understood.

There are 2 other cap or cap-associated missense variants reported with functional channel data beyond those for the gain-of-function M2225R and the loss-of-function I2270T and R2335Q reported here. The additional 2 variants are R2302H and S2195L. R2302H, like M2225R, is associated with DHS. However, in contrast to results for M2225R, *in vitro* expression studies suggest that it reduces the threshold for mechanical activation without changing the inactivation rate while also reducing surface expression through trapping of the variant channel in intracellular compartments.^22^ R2305H may therefore generate 2 conflicting effects. S2195L was found to be compound heterozygous with G253R in prune belly syndrome.^41^ It is at the interface between the cap and the outer helix (transmembrane segment 37) of the ion pore region, arguably more in the outer helix than the cap.^14^^,^^41^ Recapitulation of it in mPIEZO1 (S2211L) reduced but did not abolish mechanical sensitivity.^41^ These data are consistent with our observations, although not directly relevant to the cap, because the affected residue is not definitively in the cap. Additional cap missense variants have been identified but without information about their effects on PIEZO1 expression or function.^13^ I2270T has been detected as a compound heterozygous variant in NIFH, and so, in this case, the other variant complicates the interpretation of the effect of I2270T.^32^ This is not the case in GLD08.

The ability of Yoda1 and Yoda2b to activate the mechanically insensitive variants is important from a technical perspective because it suggests that the channels were indeed expressed and available at the cell surface membrane in our experiments. The data are also important because they suggest that mechanical insensitivity due to cap variation has the potential to be offset or overcome pharmacologically (Figure 6). Yoda1 sensitizes the channels to force,^24^ so it may left-shift force sensitivity of the variants that are otherwise out of range, in this way re-enabling force sensitivity even though Yoda1 does not interact via the cap.^55^ Such effects suggest the potential for translation to patient benefit. In support of this idea, encouraging effects of Yoda1 have occurred in WT mice. Yoda1 accelerated lymphatic valve formation, suppressed postsurgical lymphedema, restored meningeal lymphatic network function, and decreased pathological cerebrospinal fluid accumulation and ventricular enlargement in mouse models of Down syndrome.^18^^,^^25^^,^^26^^,^^42^ With such modulators, it may, for example, be possible to treat craniosynostosis,^42^ which is a clinical feature of GLD09 II.1. Therefore, PIEZO1 agonists could have a broader therapeutic value that is not limited to rescuing the function of compromised variant PIEZO1 molecules such as those described here. Lymphedemas not associated with PIEZO1 dysfunction might also be treated through PIEZO1 agonists, i.e., other primary lymphedemas and secondary lymphedemas. It is important to emphasize, however, that Yoda1 itself is probably not suitable as a therapeutic agent.^29^ We discuss this matter in the following paragraph and elsewhere.^31^

Effectiveness of PIEZO1 agonists is likely to depend on the properties of the agonists and on factors such as the availability of PIEZO1 channels and the presence of cofactors such as lymphatic flow. Determination of such factors will inform the tailoring of therapies to patients who are most likely to benefit. PIEZO1 agonists are potentially safe to administer at an appropriate dose because mice injected with Yoda1 have mostly not been adversely affected by it,^31^ and *PIEZO1* gain-of-function variants are common in some human populations and seem to be without major adverse effects.^56^^,^^57^^,^^58^ Safety will, however, depend on the specific chemistry and any off-target (i.e., non-PIEZO1) effects of each molecule tested. Understanding of PIEZO1 agonist structure-activity relationships is emerging, but the field is still in its infancy.^29^^,^^30^^,^^31^ We show the effectiveness of such agonists at a disrupted variant channel, similar to observations with prune belly syndrome variants.^41^ We also show the possibility to improve rescue through chemical modification of Yoda1 (i.e., with Yoda2b). Modifications of the left side of the molecule are promising,^29^^,^^31^ as are conservative changes to the central core (as in Yoda2b). Modifications of the right side of the molecule are also possible, although it seems to be important to maintain the integrity of the 2,6-dichlorophenyl moiety^24^^,^^28^^,^^31^ or to replace it with isosteres,^28^^,^^30^^,^^31^ as in KC124 (Figures S4 and S6A).

Variant interpretation of the G1978D and R2335Q originally suggested a VUS classification, but we show through laboratory experiments that they are disruptive, potentially enabling more accurate classification and molecular diagnosis. Under the ACMG/AMP sequence variant interpretation framework,^20^ PS3 is a criterion used to evaluate well-established *in vitro* or *in vivo* functional studies that support a damaging effect on a gene or gene product. This is a strong criterion applied when an assay demonstrates a functionally abnormal result for a variant compared to the WT; however, it can be set to a lower level of evidence and has been used at a “moderate” level here (Table 2; Table S1). All four PIEZO1 variants adversely affect the channel. Three of them reduced mechanical activation, and all showed altered responsiveness to small-molecule activators. These findings were obtained using complementary assays in hPIEZO1 or mPIEZO1: protein expression, the patch-clamp technique, which directly measures channel activity by assessing ion conduction due to mechanical activation, and the Fura-2 fluorescence assay, which evaluates free cytosolic Ca^2+^ concentration as a cellular consequence of channel activation. These assays measure distinct aspects of PIEZO1 function and provide evidence to support a PS3_moderate classification, offering moderate evidence of pathogenicity^59^ (Table 2). Nevertheless, there are complexities in the data that require further investigation such as the apparent dual loss- and gain-of-function effects of G1978D, which reduced the channel mechanical sensitivity and the channel inactivation rate. We speculate that the loss of mechanical sensitivity might be most consequential in the disease because the inactivation is not relevant if the channels are not first activated. Slower inactivation is anticipated if channels are less activated.

In summary, the results of this study suggest lymphatic relevance of PIEZO1’s mechanical sensitivity and roles of this sensitivity in GLD as well as in relatively understudied aspects of lymphatic physiology that include pericardial fluid homeostasis. The results further suggest that the PIEZO1 cap structure is critical in determining mechanical sensitivity even though the cap is not integral to the force-sensing blades. While one of the variants showed reduced expression (E829V), the other variants (I2270T, R2335Q, and G1978D) had actions on PIEZO1 that were more consistent with a loss of channel mechanical sensitivity, particularly involving the cap structure. Therefore, GLD is also associated with reduced or abolished channel (and cap) activity, which encourages the idea that some types of GLD, in which the channels are available but not physiologically active, might be treatable with PIEZO1 agonists. In an *in vitro* expression system, we demonstrate that PIEZO1 channel mechanical activation is indeed partly rescued by the PIEZO1 agonist Yoda1 and that the rescue can be improved with our Yoda1 analog, Yoda2b. Opportunities may therefore exist for improving the lives of some patients with GLD through suitable PIEZO1 agonists. This opportunity might extend to other GLD-associated variants such as L939M^11^^,^^60^ and L322P.^61^ Despite the apparently reasonable safety of Yoda1 administration in preclinical mouse studies,^18^^,^^25^^,^^26^^,^^31^^,^^42^ substantial further development and investigation of such molecules is likely to be needed before clinical trials can be considered.

### Limitations of the study

A limitation of our laboratory studies may be their dependence on overexpression of the channels in a host cell system that might generate non-physiological channel behaviors. It might be possible to overcome this limitation by recapitulating the variants in mice, as was previously achieved for PIEZO1 gain-of-function variants.^53^^,^^56^ This could, for example, be attempted for I2286T as a model of I2270T, which singularly associates with GLD (Figure 1).

## Resource availability

### Lead contact

Further information and requests for resources and reagents should be directed to and will be fulfilled by the lead contact, David J. Beech (d.j.beech@leeds.ac.uk).

### Materials availability

Plasmids, cell lines, and chemicals generated in this study are available on reasonable request.

### Data and code availability

• Data: original data for laboratory results of the main figures can be found in an Excel file that accompanies this article (Data S1).
• Code: no code was generated.
• Other items: some patient-related data may not be made available because of privacy or ethical restrictions.

## Acknowledgments

The work was supported by research grants from 10.13039/100004440Wellcome (grant no. 110044/Z/15/Z), 10.13039/501100000274British Heart Foundation (RG/17/11/33042 and SP/13/5/30288), a joint MRC/BHF program grant (MR/P011543/1 and RG/17/7/33217), and 10.13039/100016187Newlife Foundation for Disabled Children (12-13/01). Additional support was provided through studentships from 10.13039/501100000777University of Leeds (for K.C.) and the BHF (FS/4yPhD/F/20/34130 for K.A.S. and FS/18/78/33932 for E.D.). D.J.B. was supported in part by the National Institute for Health and Care Research (NIHR) Leeds Biomedical Research Centre (BRC) NIHR203331. The views expressed are those of the author(s) and not necessarily those of the NHS, the NIHR, or the Department of Health and Social Care. For the purpose of Open Access, the authors have applied a CC BY public copyright license to any Author Accepted Manuscript version arising from this submission.

## Author contributions

M.J.L. performed recombinant DNA experiments and mutagenesis, generated cell lines, designed and performed calcium measurement assays, made figures, performed data analysis, made intellectual contribution, and wrote major parts of an early draft of the manuscript. O.V.P. designed and performed patch-clamp experiments, made figures, performed data analysis, made intellectual contribution, and generated the data transparency file. D.M.L. designed and performed patch-clamp experiments, made figures, performed data analysis, and made intellectual contribution. S.M.-A. supervised the genetic studies of patients, identified the genetic variants in patients, produced information for figures and tables associated with the patient genetics and clinical features, and contributed to writing – review and editing and project administration. C.R. and K.C. designed, synthesized, and analyzed chemicals. K.A.S. performed the computer modeling and made the associated figure panels. E. Fay performed data curation and writing in the original draft. E. Fotiou performed investigation (Sanger sequencing) and formal analysis. A.B. performed investigation (seen patient in clinic) and provided resources (patients). C.H. performed investigation (seen patient in clinic) and provided resources (patients). T.L. performed investigation (seen patient in clinic) and provided resources (patients). N.B.T. performed investigation (seen patient in clinic) and provided resources (patients). S.M.W. performed investigation (seen patient in clinic) and provided resources (patients). J.C.D.R.J., E.S., and E.D. performed formal analysis (variant interpretation) and writing (review and editing). S.M. performed investigation (seen most patients in clinic) and provided resources (patients), supervised clinical staff, and contributed to writing – review and editing. G.P. provided technical assistance and contributed to writing – review and editing. A.C.K. supervised the computer modeling. R.F. supervised the chemistry and helped to generate funds. P.O. supervised the genetic studies of patients, identified the genetic variants in patients, generated associated funding, produced information for figures and tables associated with the patient genetics and clinical features, and contributed to writing – review and editing, conceptualization, resources, supervision, funding acquisition, and coordination with clinical authors. D.J.B. conceptualized the study, made intellectual contribution, supervised and orchestrated the laboratory project and team, generated funding, interpreted data, and wrote parts of the manuscript.

## Declaration of interests

D.J.B. and R.F. are partners of CalTIC GmbH, a pharmaceutical startup company with a mission to develop ion channel modulators as classes of medicines.

## STAR★Methods

### Key resources table

**Table:** 

REAGENT or RESOURCE	SOURCE	IDENTIFIER
**Antibodies**		

Horseradish peroxidase donkey anti-mouse secondary antibody	Jackson ImmunoResearch	RRID: AB_2340770
Horseradish peroxidase donkey anti-rabbit secondary antibody	Jackson ImmunoResearch	RRID: AB_10015282
Horseradish peroxidase donkey anti-rat secondary antibody	Jackson ImmunoResearch	RRID: AB_2340638
anti-HA (3F10)	Roche	Cat#12158167001; RRID: AB_390915
anti-β-actin	Santa Cruz	Cat#sc-47778; RRID: AB_626632

**Chemicals, peptides, and recombinant proteins**		

KC156	University of Leeds	Chemical structure is in Figure S4
Yoda1	Tocris	Cat#5586
Dooku1 (KC41)	University of Leeds	Chemical structure is in Figure S4
KC124	University of Leeds	Chemical structure is in Figure S4
KC146	University of Leeds	Chemical structure is in Figure S4
KC183	University of Leeds	Chemical structure is in Figure S4
CHR-1741-077	University of Leeds	Chemical structure is in Figure S4
CHR-1871-005	University of Leeds	Chemical structure is in Figure S4
Yoda2b (CHR-1871-032)	University of Leeds	Chemical structure is in Figure 3J
Lipofectamine 2000	Invitrogen	Cat#11668019
Opti-MEM	Gibco	Cat#31985062
Zeocin	InvivoGen	Cat#Ant-zn-5b
Dulbecco’s Modified Eagle’s medium	Gibco	Cat#31966-047
10% heat-inactivated fetal calf serum	Sigma-Aldrich	Cat#F9665
Penicillin Streptomycin	Sigma-Aldrich	Cat#P0781-100mL
Trizma base	Sigma-Aldrich	Cat#T6066
Sodium Chloride	ThermoFisher	Cat#S7653-250g
EGTA	Sigma-Aldrich	Cat#E4378-100g
EDTA	Sigma-Aldrich	Cat#E9884-100g
Nonidet P40 substitute	ThermoFisher	Cat#J19628.K2
Protease inhibitor cocktail	Sigma-Aldrich	Cat#P8340
Potassium Chloride	Sigma-Aldrich	Cat#P9333-500g
CaCl_2_	Honeywell	Cat#21114-1L
MgCl_2_	Honeywell	Cat# 63020-1L
Glucose	Sigma-Aldrich	Cat#G7528-250g
NaOH	ThermoFisher	Cat#S/4845/60
HEPES	Sigma-Aldrich	Cat#H4034-1kg
PrimeSTAR HS DNA Polymerase	Takara Bio	Cat#R010A
Pluronic acid (Pluronic F-127)	Sigma-Aldrich	Cat#P2443-250G
SuperSignal™ West Femto Maximum Sensitivity Substrate	ThermoFisher	Cat#34095
Molecular Probes™ Fura-2 a.m.	Invitrogen	Cat#F1201
DMSO	Honeywell	Cat#D5879-100ML

**Deposited data**		

Refseq database of UCSC^62^	NCBI	https://www.ncbi.nlm.nih.gov/refseq/
Protein DataBank (PDB:6B3R)	RCSB	https://www.rcsb.org/structure/6B3R

**Experimental models: Cell lines**		

T-REx-293 cell line; The cell line was not independently validated by our laboratory	Invitrogen	Cat#R71007
HEK-T-Rex mPiezo1 (stable cell line)	This paper	N/A
HEK-T-Rex mPiezo1 (with HA tag) (stable cell line)	This paper	N/A
HEK-T-Rex mPiezo1 I2286T (stable cell line)	This paper	N/A
HEK-T-Rex mPiezo1 R2351Q (stable cell line)	This paper	N/A
HEK-T-Rex mPiezo1 G1994D (stable cell line)	This paper	N/A
HEK-T-Rex mPiezo1 E824V (stable cell line)	This paper	N/A
HEK-T-Rex hPiezo1 (transiently expressed)	This paper	N/A
HEK-T-Rex hPiezo (with HA tag) (transiently expressed)	This paper	N/A
HEK-T-Rex hPiezo1 E829V (transiently expressed)	This paper	N/A
HEK-T-Rex hPiezo1 G1978D (transiently expressed)	This paper	N/A
HEK-T-Rex hPiezo1 R2335Q (transiently expressed)	This paper	N/A
HEK-T-Rex hPiezo1 I2270T (transiently expressed)	This paper	N/A

**Oligonucleotides**		

hPiezo1-HA hPiezo forward primer GGTCTACCTGCTCTTCCTGCTG	This paper	N/A
hPiezo1-HA reverse primer incorporating a ‘ASA’ linker and the HA sequence CCCATACGATGTTCCAGATTACGCTTA GGCGACTCTAGATCATAATCAGCCATACC	This paper	N/A
hPiezo1-HA vector (pcDNA™4/TO) forward primer CCCATACGATGTTCCAGATTACGCT TAGGCGACTCTAGATCATAATCAGCCATACC	This paper	N/A
hPiezo1-HA reverse primer CAGCAGGAAGAGCAGGTAGACC	This paper	N/A
mPiezo1-HA Overlapping mPIEZO1 forward primer GTAACAACTCCGCCCCATTG	This paper	N/A
mPiezo1-HA reverse primers CTAAGCGTA ATCTGGAACATCGTATGGGTACTCCCTCTCACGT	This paper	N/A
mPiezo1-HA vector (pcDNA™4/TO) forward primer CATACGATGTTCCAGATTACGCTT AGCCGCTGATCAGCCTCG	This paper	N/A
mPiezo1-HA vector (pcDNA™4/TO) reverse primer CAATGGGGCGGAGTTGTTAC	This paper	N/A

**Recombinant DNA**		

Invitrogen V102020 pcDNA™4/TO Mammalian Expression Vector	fisher scientific	Cat#V102020
pcDNA4/TO-mPIEZO1 constructs	This paper	N/A
Human PIEZO1_AcGFP	University of Leeds	N/A
pcDNA3_mouse PIEZO1_IRES_GFP	Ardem Patapoutian laboratory	Addgene Plasmid #80925
Primer sequences to generate Piezo1 variants: see Table S2	This paper	N/A

**Software and algorithms**		

pClamp (v10.6) software	Molecular Devices, USA	https://moldevkb.blob.core.windows.net/kb01/software/cns/pclamp/10/pCLAMP_10_6_2.exe
MODELER (v9.19)	Salilab	https://salilab.org/modeller/9.19/release.html
PyMOL	Schrödinger	https://www.pymol.org/
Origin^R^2018 software	OriginLab Corporation, USA	https://www.originlab.com/index.aspx?go=Support&pid=3301
REVEL	N/A	https://sites.google.com/site/revelgenomics/
AlphaMissense	N/A	https://github.com/google-deepmind/alphamissense
CADD v1.7	N/A	https://cadd.gs.washington.edu/
SpliceAI	N/A	https://github.com/Illumina/SpliceAI
GnomAD v4	N/A	https://gnomad.broadinstitute.org/
Clinvar	N/A	https://www.ncbi.nlm.nih.gov/clinvar/

### Experimental model and study participant details

#### Patient ascertainment

3 individuals with a GLD phenotype and available family members were included in the study. All are external referrals from clinicians to the St. George’s clinical academic research team. Genomic DNA was analyzed for sequence variants in all exons of *PIEZO1* by Sanger sequencing as described previously.^11^ Samples of available family members were subsequently analyzed by Sanger sequencing for the variants identified in their respective proband. Findings were subsequently confirmed in a molecular diagnostics laboratory. Single-letter amino acid codes are used to refer to the effects of gene variants on the amino acid sequence of the protein.

### Ethics approval statement and permission to publish

Ethical approval for this study was obtained from the South West London Research Ethics Committee (REC ref. 05/Q0803/257), and written informed consent was obtained from all participants. Permission to publish was also obtained.

#### Clinical information on the GLD patients

##### GLD07

Proband (GLD07 II.1) is a 13-year-old male born to non-consanguineous parents. First trimester nuchal translucency was normal. Antenatal imaging (gestational date not noted) identified NIFH and polyhydramnios. The baby was hydropic at birth (Figure 1B) which resolved spontaneously. However, over a period of 6–12 months, he developed symmetrical lower limb lymphedema with intermittent upper limb, facial (eyelid) and genital (scrotal) lymphedema. At the age of two years his peripheral edema had stabilised mainly affecting the feet, with persistent pleural effusions and minimal perihepatic ascites, diagnosed on MR imaging. Apart from a maldescended left testis, he has no other structural malformations. He has micrognathia and slight facial swelling but is otherwise non-dysmorphic. There are no concerns about his neurodevelopment. At the age of three years, he developed cellulitis (followed by rheumatic fever) which was probably lymphedema-related and he has been on penicillin prophylaxis since.

##### GLD08

The proband (GLD08 II.1) is a 30-year-old male, born to consanguineous (first cousins once removed) parents from Pakistan. His antenatal and neonatal period was uneventful. He was born at term with a normal birth weight of 3370g. From age 11 years he developed progressive breathlessness and reduced exercise tolerance and was found to have bilateral chylous pleural effusions (triglycerides 19.42mmol/L with moderate lymphocytosis). Treatment has proved very difficult and has involved pleurodesis, ligation of the thoracic duct and pleuro-peritoneal shunting. He was also found to have a hydrocele (likely due to a patent ductus vaginalis) and ascites. At 17 years old he developed a pericardial effusion that rapidly progressed to cardiac tamponade. Pericardiocentesis confirmed chylous pericardial fluid, and this was repeated less than 3 weeks later due to recurrence of the pericardial effusion and tamponade (Figure 1B). He was subsequently treated with octreotide and medium chain triglyceride diet for two years. He has residual, significant and symptomatic restrictive lung disease requiring nocturnal non-invasive ventilation. He developed bilateral lower limb lymphedema with scrotal edema some years later at the age of 30 years. Additional clinical features include mild microcephaly, short stature, and generalised osteopenia. He has a normal male karyotype and a 12-gene Ras-MAPK pathway disorders panel identified no causal variants. Lymphoscintigraphy has not been performed.

GLD08 I.2 is the proband’s father. His parents are not known to be consanguineous. He was diagnosed with spontaneous bilateral chylous effusions at the age of 30 years. Despite pleurodesis, his effusions are persistent resulting in a restrictive lung defect and nocturnal hypoventilation requiring non-invasive ventilation (NIV). He also has a mild-moderate pericardial effusion, which has been stable and has not required pericardiocentesis. He has bilateral lower limb pitting edema to the knees (onset at the age of 47 years) and varicose veins with varicose eczema at the ankles. He has no further complications and is developmentally normal. Venous duplex and lymphoscintigraphy not performed.

The proband’s brother, GLD08 II.2, was found to have bilateral pleural effusions at the age of 12 years. A left drain was placed and chyle confirmed (triglycerides 21 mmol/L in pleural fluid). He also presented with mild peripheral edema of the lower limbs, and tense bilateral hydrocoeles. In addition, he also had truncal obesity, intellectual disability, unilateral post-axial polydactyly of one foot and a micropenis related to his co-existing diagnosis of Bardet Biedl Syndrome.

Proband’s maternal aunt (GLD08 I.1) had progressive pleural and pericardial effusions (with pleural and pericardial thickening) that first started aged 15 years. She died from chylothoraces and pleural disease aged 33 years and was not formally assessed or tested in clinic.

##### GLD09

GLD09 II.1 is the first child of non-consanguineous Caucasian parents, conceived by *in vitro* fertilisation. The first trimester nuchal translucency measurement was normal. At 19-week’ gestation the baby was noted to have bilateral hydrothoraces and facial edema. Amniocentesis at this time revealed a 46,XY karyotype. The pregnancy was complicated by polyhydramnios requiring repeated amnio-drainage at 27, 30 and 33 weeks. A male infant was born at 34-week’ gestation with significant facial swelling and bilateral chylothoraces requiring bilateral chest drains (Figure 1B). He was intubated and ventilated for one month and remained in neonatal intensive care for a total of three months. During this period, he had laryngomalacia and severe gastro-oesophageal reflux that required a fundoplication at 7 months of age. A gastrostomy was inserted owing to an oral aversion, with a low fat MCT diet instituted in the setting of the bilateral chylothoraces. A lymphoscintigram at 9 months demonstrated extensive subdermal flow of lymphatics in the lower limbs, chest wall and scrotum. Upon review at 2 years of age, he had edema of the lower limbs to the thighs, and genitalia, as well as intermittent facial swelling (Figure 1B). He had metopic synostosis and a facial haemangioma over the glabella. He had a circumcision at 2½ years of age for significant scrotal/genital edema. By 7 years of age he had contracted pneumonia on three occasions and was hospitalised with invasive group A Streptococcal septicaemia. He also began to exhibit bilateral periorbital and conjunctival vascular changes with small punctate haemorrhages. He had obstructive sleep apnoea from age 7 years treated with nocturnal CPAP. Orthodontic treatment with an upper jaw expander has resulted in clinical improvement of sleep apnoea symptoms. Early concerns regarding speech and language delay were resolved by the commencement of primary school. He was diagnosed with Asperger syndrome at 3 years of age. There is a bi-lineal family history of autism spectrum disorder.

GLD09 I.1 is the proband’s father. He has four limb lymphedema, with swelling of the lower limbs at birth, followed by swelling of the hands from the age of 24 years. The cause of this is unknown. He has had no episodes of cellulitis and no history of pleural effusions. He also has a diagnosis of Asperger syndrome. He only has one variant in *PIEZO1* [c.2486A>T; p.(E829V)].

Further information about the patients can be found in Table 1 and Figure 1.

#### *PIEZO1* variants *in silico* analysis

All the relative genomic and protein positions of PIEZO1 reported here correspond to the transcript PIEZO1-001 (RefSeq: NM_001142864, Ensembl: ENST00000301015.9) and Q92508 Uniprot protein accession ID, respectively. The reported genomic coordinates refer to the GRCh38/hg38 human genome reference. Changes in the gene structure and/or amino acid sequence, due to the reported variants, were retrieved by the Refgene database of UCSC.^62^ The Allele Frequencies (AF) of the reported variants were checked in gnomAD databases^63^ and their pathogenicity was predicted by the Combined Annotation Dependent Depletion (CADD) tool^64^ (Table S1).

#### PIEZO1 constructs

Human PIEZO1_AcGFP^65^ was used as a template to clone the human PIEZO1 (hPIEZO1) sequence with a C-terminal HA-epitope. Overlapping hPIEZO1 (forward primer 5′ GGTCTACCTGCTCTTCCTGCTG 3′) and reverse primer incorporating an ‘ASA’ linker and the HA sequence 5′ CCCATACGATGTTCCAGATTACGCTTAGGCGACTCTAGATCATAATCAGCCATACC 3′) and vector (forward primer 5′ CCCATACGATGTTCCAGATTACGCTTAGGCGACTCTAGATCATAATCAGCCATACC 3′ and reverse primer 5′ CAGCAGGAAGAGCAGGTAGACC 3′) PCR products were assembled using Gibson Assembly. Missense variants were introduced by site-directed mutagenesis (PCR primer sequences are provided in Table S2).

pcDNA3_mouse PIEZO1_IRES_GFP, a gift from A Patapoutian, was used as a template to clone the mouse PIEZO1 (mPIEZO1) coding sequence with a C-terminal HA-epitope, into pcDNA4/TO. Overlapping mPIEZO1 (forward primer 5′ GTAACAACTCCGCCCCATTG 3′ and reverse primers 5′ CTAAGCGTAATCTGGAACATCGTATGGGTACTCCCTCTCACGT 3′) and pcDNA4/TO (forward primer 5′ CATACGATGTTCCAGATTACGCTTAGCCGCTGATCAGCCTCG 3′ and reverse primer 5′ CAATGGGGCGGAGTTGTTAC 3′) PCR products (PrimeSTAR HS DNA Polymerase, TaKaRa) were assembled using Gibson Assembly (NEB). Missense variants were introduced into mPIEZO1 with and without HA-epitope by site-directed mutagenesis (PCR primer sequences are in Table S2).

#### Cell culture

HEK 293 cell line (female origin) was transiently transfected at 90% confluence using Lipofectamine 2000 (Invitrogen) in OptiMEM (Gibco). Briefly: 500 ng endotoxin-free DNA per 100μL OptiMEM was prepared alongside 3μL Lipofectamine 2000 per 100μL OptiMEM (Gibco). Both were incubated for 5 min and then mixed, and then incubated at room temperature (21°C–24°C) for 20–30 min. Finally, the transfection mix was gently added dropwise to the cells and the cells were placed in a humidified incubator at 37°C supplied with 5% CO_2_ for 5 h. Medium was replaced afterward, and cells were used for experimentation 48 h after transfection.

T-REx-293 cell line was transfected with pcDNA4/TO-mPIEZO1 constructs using Lipofectamine 2000 (Invitrogen) as above and treated with 200 μg.mL^−1^ zeocin (InvivoGen) to select for stably transfected cells. At least two strongly expressing clonal cell lines were established and tested for each variant. All cell lines were maintained in Dulbecco’s Modified Eagle’s medium (Invitrogen) supplemented with 10% heat-inactivated fetal calf serum (Sigma-Aldrich), penicillin (50 units.mL^−1^) and streptomycin (0.5 mg.mL^−1^) (Sigma-Aldrich) and grown at 37°C in a humidified 5% CO_2_ incubator.

Cells were routinely checked for myoplasma contamination and confirmed to be mycoplasma-free.

#### Western blotting

For western blotting, cells were harvested in lysis buffer (10 mM Tris, pH 7.4, 150 mM NaCl, 0.5 mM EDTA, 0.5% Nonidet P40 substitute) containing protease inhibitor cocktail (Sigma-Aldrich). Equal protein amounts were loaded on 7% polyacrylamide gels and resolved by electrophoresis. Samples were transferred to PVDF membranes and labeled overnight with anti-HA (0.01 μg.mL^−1^, Roche clone 3F10), anti-β-actin (200 ng.mL^−1^, Santa Cruz). Horseradish peroxidase donkey anti-mouse/rabbit/rat secondary antibodies (1:10000, Jackson ImmunoResearch) and SuperSignal Femto detection reagents (Pierce) were used for visualisation.

#### Electrophysiology and mechanical stimulation

Ionic currents were recorded through outside-out patches from cells using standard patch-clamp techniques in voltage-clamp mode. Patch pipettes were fire-polished and had resistance of 4–7 MΩ when filled with pipette solution. Ionic solution of composition (mM) NaCl 140, HEPES 10 and EGTA 5 (titrated to pH 7.4 using NaOH) was used in both the pipette and bath. Recordings were at a constant holding potential of −80 mV. 200-ms pressure steps were applied to the patch pipette with an interval of 12 s using High Speed Pressure Clamp HSPC-1 System (ALA Scientific Instruments, USA). All recordings were made with an Axopatch-200B amplifier (Axon Instruments, Inc., USA) equipped with Digidata 1550B and pClamp (v10.6) software (Molecular Devices, USA) at room temperature (21 ± 2°C). Currents were filtered at 2–5 kHz and digitally sampled at 5–20 kHz.

#### Intracellular Ca^2+^ measurement

For intracellular Ca^2+^ assays, cells were plated at 80–90% confluence in 96-well plates 24 h prior to recordings (6–8 × 10^4^ cells per well). To measure intracellular Ca^2+^, cells were incubated for 1 h at 37°C in standard bath solution (SBS) of composition 135 mM NaCl, 5 mM KCl, 1.2 mM MgCl_2_, 1.5 mM CaCl_2_, 8 mM glucose and 10 mM HEPES (pH titrated to 7.4 using NaOH) containing 2 μM fura-2-acetoxymethyl ester (fura-2-AM, Molecular Probes) with 0.01% weight/volume pluronic acid. Cells were washed with SBS and incubated at room temperature for 20 min prior to recordings. Equal volumes of 2x concentrated compounds were injected to test for acute channel activation. Pre-incubation with compounds occurred during the 20 min prior to recordings. To expose cell membranes to hypo-osmolality, an equal volume of hypotonic SBS (containing only 35 mM NaCl) was injected onto the cells. Measurements were made on a fluorescence plate reader (Flexstation III, Molecular Devices) at room temperature (21 ± 2°C). Fura-2 was excited at 340 nm and 380 nm and emitted light collected at 510 nm, with measurements shown as the change in fluorescence (F) ratio (ΔF_340/380_).

#### Molecular modeling

Structural data for mPIEZO1 were obtained at Protein DataBank (PDB:6B3R). Missing loop regions were modeled using MODELER (v9.19). Large intracellular loops (located at residues 718–781, 1366–1492, 1579–1654 and 1808–1951) remained unstructured following modeling and were removed from the final model. A structural model of hPIEZO1 was generated based on the mPIEZO1 model.

#### Chemistry

KC41 (Dooku1) was prepared as described.^28^ All purchased chemicals and solvents were used without further purification unless otherwise stated. All compounds were at least 95% pure by 1H NMR. 1H Nuclear Magnetic Resonance spectra were recorded at 500 MHz using a Bruker DRX 500 instrument or at 400 MHz using a Bruker DPX 400. 1H spectra are referenced based on the residual proton in the solvent (e.g., the CHCl_3_, 0.01% in 99.99% CDCl_3_). Coupling constants (*J*) are reported to the nearest 0.1 Hz. 13C NMR spectra were recorded at 125 MHz on 500 MHz spectrometers or at 100 MHz on 400 MHz spectrometers. LC-MS was performed on a Bruker Daltronics running a gradient of increasing acetonitrile (5–95%) in H_2_O both containing 0.1% formic acid at 1 mL.min^−1^, on a short path C18 reverse phase column, detecting compounds with both a diode array detector and a Bruker mass spectrum analyser. HRMS was performed on a Bruker Daltonics micrOTOF using positive electrospray ionisation (ES+). Automated column chromatograph was carried out using a Biotage Isolera Four, using either Sfär Silica D or KP-Silica cartridges. HPLC was performed on an Agilent 1290 Infinity Series equipped with a UV detector and Hyperprep C18 reverse phase column. Key to NMR abbreviations: s (singlet), br s (broad singlet) d (doublet), dd (doublet of doublets), ddd (doublet of doublets of doublets), t (triplet), dt (doublet of triples), q (quartet), m (multiplet), ap. t (apparent triplet). For experiments, Yoda1 and its analogues were prepared as 10 mM stock solutions in 100% dimethyl sulfoxide (DMSO) before serial dilution in DMSO and final dilution in aqueous solution. Cells were exposed to 0.1% DMSO for all concentrations of the compounds from 0 to 10 μM.

Aqueous solubility, mouse microsomal stability and mouse plasma protein binding assays were performed by Malvern Panalytical (UK) as previously described.^29^

Scheme 1 (Figure S9) Reagents: a. 2,6-dichlorobenzyl chloride, K_2_CO_3_, DMF, 90°C, N_2_, 1h, 83% b. NBS, DMF, 3h, 73% c. 4-methoxycarbonylphenyl boronic acid, 2M Na_2_CO_3 (aq)_, Pd(PPh_3_)_4_, 1,4-dioxane, 90°C, N_2_, 18h, 38% d. 10M NaOH _(aq)_, THF, RT-60°C, 16.5 h, 69% e. 2,6-dimethylbenzyl chloride, KOH, DMF, RT-80°C, N_2_, 2h, 23% f. Thiourea, EtOH, reflux, 45 min, 99% g. 2M NaOH _**(aq**)_, EtOH, reflux, N_2_, 5h, then 1M HCl, RT, N_2_, 18 h, 71% h. 2-bromo-1,3,4-thiadiazole, K_2_CO_3_, DMF, 90°C, N_2_, 18 h, 57% i. NBS, DCM, reflux, 48h, 78% j. Appropriate phenyl boronic acid, K_2_CO_3_, Pd(PPh_3_)_4_, 1,4-dioxane, H_2_O, 90°C, N_2_, 2-24h, 18–25% k. Et_3_N, DMF, 90°C, N_2_, 90^o^C, 21h, 80% l. piperidine, EtOH, reflux, N_2_, 21h, 39% m. Et_3_N, DMF, 90^o^C, N_2_, 20h, 74% n. isoxazole-5-carbonyl chloride, Et_3_N, DMAP, THF, RT, 20 h, 67%.

#### General procedure A

The desired aromatic halide (1.0 eq.) the desired boronic acid/ester (1–3.5 eq.) and K_2_CO_3_ (4.0 eq.) were dissolved in anhydrous 1,4-dioxane (2 mL) and H_2_O (2 mL) then degassed with N_2_ for 30 min. Pd(PPh_3_)_4_ (0.15 eq.) was then added and the reaction was then heated to 90°C for 2–24 h. Upon completion, the reaction was diluted with H_2_O (20 mL), extracted with DCM (3 × 10 mL), dried over Na_2_SO_3_, filtered and reduced in vacuo to afford the crude or pure product.

##### 2-([(2,6-dichlorophenyl)methyl]sulfanyl)-1,3-thiazole (**1**)

To a solution of 1,3-thiazole-2-thiol (134 mg, 1.14 mmol) and K_2_CO_3_ (170 mg, 1.05 mmol) in DMF (4 mL) under N_2_ was added 2,6-dichlorobenzyl chloride (240 mg, 1.05 mmol) and the reaction heated to 90°C. After 1h the reaction was cooled to RT and diluted with H_2_O (70 mL). The aqueous suspension was extracted with EtOAc (3 × 70 mL) and the combined organic phases were washed with brine (50 mL) and 10% LiCl solution (30 mL), dried (Na_2_SO_4_) and concentrated *in vacuo*. Crude mixture purified by ACC (0–10% EtOAc in Petroleum ether (40°C–60°C)) to isolate the compound as a colourless liquid (260 mg, 0.94mmol, 83%). Rf 0.31 (19:1 Petroleum ether (40°C–60°C):EtOAc (v/v)); δ_H_ (400 MHz, CDCl_3_): 7.76 (1H, d, thiazole H-4, *J* = 3.4 Hz), 7.31-7.29 (3H, m, thiazole H-5 and benzyl H-3/5), 7.16 (1H, ap. t, benzyl H-4, *J* = 6.6 and 8.4 Hz) 4.75 (2H, s, benzylic CH_2_); δ_C_ (100 MHz, CDCl_3_); 162.7 (thiazole C-2), 143.2 (thiazole C-4), 136.1 (benzyl C-2/6), 132.6 (benzyl C-1), 129.4 (benzyl C-4), 128.4 (benzyl C-3/5) 120.6 (thiazole C-5), 35.4 (benzylic CH_2_); m/z ES + Found MH^+^ 275.9462, C_10_H_8_Cl_2_NS_2_ requires MH^+^ 275.9469.

##### 5-Bromo-2-([(2,6-dichlorophenyl)methyl]sulfanyl)-1,3-thiazole (**2**)

To a solution of 2-([(2,6-dichlorophenyl)methyl]sulfanyl)-1,3-thiazole (**1**) (100 mg, 0.36 mmol) in DMF (3 mL) was added *N*-bromosuccinimide (97 mg, 0.54 mmol) and the reaction stirred at RT. After 3h the reaction was diluted with H_2_O (50 mL) and the aqueous solution extracted with EtOAc (3 × 30 mL). The combined organic phases were washed with brine (50 mL) and 10% LiCl solution (50 mL), dried (Na_2_SO_4_) and concentrated *in vacuo*. The crude product was purified by ACC (0–10% EtOAc in Petroleum ether (40°C–60°C)) to isolate the compound as a colourless liquid (93 mg, 0.26mmol, 73%). Rf 0.43 (24:1 Petroleum ether (40°C–60°C):EtOAc (v/v)); δ_H_ (400 MHz, CDCl_3_): 7.55 (1H, s, thiazole H-4), 7.24 (2H, d, benzyl H-3/5, *J* = 8.1Hz), 7.11-7.07 (1H, m, benzyl H-4), 4.62 (2H, s, benzylic CH_2_); δ_C_ (100 MHz, CDCl_3_); 163.6 (thiazole C-2), 144.4 (thiazole C-4), 136.0 (benzyl C-2/6), 132.4 (benzyl C-1), 129.5 (benzyl C-4), 128.5 (benzyl C-3/5), 109.0 (thiazole C-5), 35.4 (benzylic CH_2_); LC-MS m/z ES + Found MH+ 355.78.

##### Methyl 4-(2-([(2,6-dichlorophenyl)methyl]sulfanyl)-1,3-thiazol-5-yl)benzoate (**3**)

A solution of 5-bromo-2-([(2,6-dichlorophenyl)methyl]sulfanyl)-1,3-thiazole (**2**) (93 mg, 0.26 mmol), 4-methoxycarbonylphenyl boronic acid (57 mg, 0.31 mmol) and 2M Na_2_CO_3_ (0.39 mL, 0.79 mmol) in 1,4-dioxane (4 mL) under N_2_ was degassed for 20 min and Pd(PPh_3_)_4_ (15 mg, 0.01 mmol) added. The reaction was heated to 90°C for 18 h, then cooled to RT and diluted with H_2_O (50 mL). The aqueous solution was extracted with EtOAc (2 × 40 mL) and the combined organic phases washed with brine (50 mL), dried (Na_2_CO_3_) and concentrated *in vacuo*. The crude product was purified using ACC (0–10% EtOAc in Petroleum ether (40°C–60°C), then 0–25% EtOAc in Petroleum ether (40°C–60°C)) to isolate the desired compound as a yellow solid (41 mg, 0.10 mmol, 38%). Rf 0.45 (4:1 Petroleum ether (40°C–60°C):EtOAc (v/v)); δ_H_ (400 MHz, CDCl_3_): 7.98 (2H, d, C*H*CCOOMe, *J* = 8.4 Hz), 7.91 (1H, s, thiazole H-4), 7.49 (2H, d, C*H*C-thiazole, *J* = 8.4 Hz), 7.25 (2H, d, benzyl H-3/5, *J* = 8.1 Hz), 7.12-7.09 (1H, m, benzyl H-4), 4.72 (2H, s, benzylic CH_2_), 3.86 (3H, s, CO_2_*Me*); δ_C_ (100 MHz, CDCl_3_); 166.4 (*C*O_2_Me), 163.5 (thiazole C-2), 139.6 (thiazole C-5), 139.3 (thiazole C-4), 136.1 (benzyl C-2/6), 135.4 (C-thiazole), 132.4 (benzyl C-1), 130.5 (*C*CO_2_Me), 129.6 (*C*HCCO_2_Me), 129.5 (benzyl C-4), 128.5 (benzyl C-3/5), 126.2 (*C*HC-thiazole), 52.2 (CO_2_*Me*) 35.2 (benzylic CH_2_); m/z ES + Found MH^+^ 409.9832, C_18_H_14_Cl_2_NO_2_S_2_ requires MH^+^ 409.9837.

##### 4-(2-([(2,6-Dichlorophenyl)methyl]sulfanyl)-1,3-thiazol-5-yl)benzoic acid **CHR-1871-032**

To a solution of methyl 4-(2-([(2,6-dichlorophenyl)methyl]sulfanyl)-1,3-thiazol-5-yl)benzoate (**3**) (34 mg, 0.08 mmol) in THF (4 mL) was added 10 M NaOH aq. solution (0.04 mL, 0.41 mmol) and the reaction stirred at RT for 1h then heated to 60°C for 10 h. Further 10 M NaOH aq. solution (0.04 mL, 0.41 mmol) added and the reaction heated for a further 8 h before stirring at RT for 2 days. Further 10 M NaOH aq. solution (0.04 mL, 0.41 mmol) added and the reaction heated at 60°C for a further 6.5h. The reaction was then cooled to RT and the volatile solvents removed *in vacuo*. The crude mixture was diluted with H_2_O (20 mL) and extracted with EtOAc (3 × 20 mL). The aqueous solution was acidified with 1 M HCl to pH1 and the resulting precipitate collected by filtration isolating 4-(2-([(2,6-dichlorophenyl)methyl]sulfanyl)-1,3-thiazol-5-yl)benzoic acid as a pale yellow solid (22 mg, 0.06 mmol, 69%). δ_H_ (500 MHz, d_6_-DMSO): 13.09 (1H, br. s, CO_2_H), 8.38 (1H, s, thiazole H-4), 7.99 (2H, d, *J* = 8.4 Hz, C*H*CCO_2_H), 7.77 (2H, d, *J* = 8.4 Hz, phenyl H-2/6), 7.55 (2H, d, *J* = 8.1 Hz, benzyl H-3/5), 7.42-7.39 (1H, m, benzyl H-4), 4.76 (2H, s, benzylic CH_2_); δ_C_ (125 MHz, d_6_-DMSO): 167.2 (thiazole C-2), 162.3 (CO_2_H), 141.1 (thiazole C-4), 139.5 (thiazole C-5), 135.5 (phenyl C-1), 134.9 (benzyl C-2/6), 132.4 (phenyl C-4), 131.1 (benzylic C-4), 130.7 (phenyl C-3/5), 129.3 (benzylic C-3/5), 126.7 (phenyl C-2/6), 35.2 (benzylic CH_2_); m/z ES + Found MH^+^ 395.9677, C_17_H_12_Cl_2_NO_2_S_2_ requires MH^+^ 395.9681.

##### 2-((2,6-Dimethylbenzyl)thio)-5-(pyrazin-2-yl)-1,3,4-thiadiazole **KC124**

5-(2-Pyrazine)-1,3,4-thiadiazole-2-thiol (25 mg, 0.13 mmol) and KOH (8 mg, 0.15 mmol) were added to degassed DMF (5 mL) under a flow of nitrogen. This solution was stirred for 1 h followed by dropwise addition of 2,6-dimethylbenzyl chloride (22 mg, 0.14 mmol) in degassed DMF (2 mL) over 25 min The solution was heated to 80°C and allowed to stir for 2 h. The reaction mixture was then diluted with H_2_O (20 mL) and extracted with EtOAc (3 × 15 mL), the organic layers were combined and washed with sat. NH_4_Cl solution (2 × 15 mL), sat. NaHCO_3_ solution (2 × 15 mL), sat. NaCl solution (2 × 15 mL), 10% LiCl solution (w/w) (2 × 15 mL), dried over Na_2_SO_4_, filtered and evaporated to dryness *in vacuo*. This was purified by ACC (0–80% EtOAc in Petroleum ether (40°C–60°C)) to afford a white powder (10 mg, 0.03 mmol, 23%). Rf 0.45 (7:3 Petroleum ether (40°C–60°C):EtOAc (v/v)); δ_H_ (400 MHz, CDCl_3_): 9.48 (1H, d, J = 1.5 Hz, pyrazinyl 3-H), 8.58 (1H, d, *J* = 2.5 Hz, pyrazinyl 6-H), 8.53-8.52 (1H, m, pyrazinyl 5-H), 7.06 (1H, dd, J = 8.5 & 6.5 Hz, benzyl 4-H), 6.99 (2H, d, *J* = 7.5 Hz, benzyl 3-H), 4.70 (2H, s, benzyl CH_2_), 2.39 (6H, S, Me); δ_C_ (100 MHz, CDCl_3_); 169.1 (thiadiazole 5-C), 167.4 (thiadiazole 2-C), 145.7 (pyrazinyl 6-C), 144.8 (pyrazinyl 2-C), 144.2 (pyrazinyl 5-C), 142.4 (pyrazinyl 3-C), 137.9 (benzyl 4-C), 130.9 (benzyl 1-C), 128.5 (benzyl 3-C), 128.3 (benzyl 3-C), 33.6 (benzyl CH_2_), 19.7 (Me); m/z ES + Found MH^+^ 337.0525, C_15_H_14_N_4_S_2_ requires MH^+^ 337.0558.

##### 2,6-dichlorophenyl)methyl carbamimidothioate hydrochloride (**4**)

A solution of 2,6-dichlorobenzyl chloride (921 mg, 4.71 mmol) and thiourea (362 mg, 4.76 mmol) in EtOH (15 mL) was heated to reflux for 45 min. The reaction mixture was cooled to RT and concentrated *in vacuo* to give the title compound as a white solid (1.27 g, 4.68 mmol, 99%). Used without further purification. Compound can be stored for several months. δ_H_ (400 MHz, d_6_-DMSO): 9.52 (4H, s, 2 × NH_2_), 7.58 (2H, d, benzyl H-3/5, *J* = 8.2 Hz) 7.47-7.43 (1H, m, benzyl H-4), 4.70 (2H, s, benzylic CH_2_); δ_C_ (100 MHz, d_6_-DMSO): 169.7 (S*C*(NH_2_)NH_2_), 135.6 (benzyl C-2/6), 131.8 (benzyl C-1), 130.2 (benzyl C-4), 129.4 (benzyl C-3/5), 32.0 (benzylic CH_2_); m/z ES + Found MH^+^ 234.9848, C_8_H_9_Cl_2_N_2_S requires MH^+^ 234.9858.

##### 2,6-Dichlorobenzyl thiol (**5**)

A solution of (2,6-dichlorophenyl)methyl carbamimidothioate hydrochloride (**4**) (797mg, 3.93 mmol) in EtOH (15 mL) under N_2_ was treated with 2 M aq. NaOH (5.9 mL, 11.8 mmol) and heated to reflux. After 5 h the reaction was cooled to RT and 1 M HCl (15.7 mL, 15.7 mmol) added. The reaction was stirred for 18h, then diluted with H_2_O (80 mL). The aqueous solution was extracted with EtOAc (3 × 70 mL) and the combined organic phases washed with brine (70 mL), dried over MgSO_4_ and concentrated *in vacuo* to give 2,6-dibenzyl thiol as a colourless oil which forms a white solid on standing (535 mg, 2.77 mmol, 71%. Compound must be used within weeks of synthesis. δ_H_ (400 MHz, CDCl_3_): 7.23 (2H, d, benzyl H-3/5, *J* = 8.04 Hz), 7.07-7.03 (1H, m, benzyl H-4), 3.92 (2H, d, benzyl CH_2_, *J* = 8.4 Hz), 2.02 (1H, t, SH, *J* = 8.4 Hz); δ_c_ (100 MHz, CDCl_3_): 137.3 (C-2/6), 134.6 (C-1), 129.0 (C-4), 128.4 (C-3/5), 24.4 (benzyl CH_2_).

##### 2-((2,6-Dichlorobenzyl)thio)-1,3,4-thiadiazole (**6**)

2,6-dichlorobenzyl thiol (**5**) (2.85 g, 14.77 mmol), 2-bromo-1,3,4-thiadiazole (2.43 g, 14.77 mmol), & K_2_CO_3_ (2.38 g, 17.72 mmol) were dissolved in DMF (10 mL) and heated to 90°C for 18 h. The reaction was diluted with H_2_O (100 mL), extracted with EtOAc (3 × 40 mL) and the organic layers combined. These were washed with brine (3 × 40 mL), 10% LiCl (3 × 40 mL), dried over MgSO_4_, filtered and concentrated *in vacuo* to give brown residue (4.33 g). This was purified by ACC (0–30% EtOAc in petroleum ether (40°C–60°C)) to afford white crystalline solid (2.33 g, 8.43 mmol, 57%) Rf 0.60 (7:3 Petroleum ether (40°C–60°C):EtOAc (v/v)); δ_H_ (400 MHz, CDCl_3_): 8.99 (1H, s, thiadiazole 5-H), 7.27 (2H, d, *J* = 8.5 Hz, benzyl 3-H), 7.13 (1H, t, *J* = 8.5 H, benzyl 4-H), 4.89 (2H, s, benzyl CH_2_); δ_C_ (100 MHz, CDCl_3_): 164.7 (thiadiazole 2-C), 152.1 (thiadiazole 5-C), 136.3 (benzyl 2-C), 131.7 (benzyl 1-C), 129.8 (benzyl 4-C), 128.5 (benzyl 3-C), 34.73 (benzyl CH_2_); m/z ES + Found MNa^+^ 298.9235, C_9_H_6_Cl_2_N_2_S_2_ requires MNa^+^ 298.9242.

##### 2-Bromo-5-((2,6-dichlorobenzyl)thio)-1,3,4-thiadiazole (**7**)

2-((2,6-dichlorobenzyl)thio)-1,3,4-thiadiazole (**6**) (2.33 g, 8.43 mmol), & *N*-bromosuccinimide (2.10 g, 11.80 mmol) were dissolved in DCM (10 mL) and refluxed for 48 h. The reaction was then cooled, quenched with sat. aq. Na_2_S_2_O_3_ (20 mL), partitioned and the aqueous extracted with DCM (2 × 15 mL). The organic layers were combined, dried over MgSO_4_, filtered and concentrated *in vacuo* to give an orange, oily crystals (3.15 g). This was purified by ACC (0–30% EtOAc in Petroleum ether (40°C–60°C)) to afford a white crystalline solid (2.80 g, 7.87 mmol, 78%) Rf 0.80 (7:3 Petroleum ether (40°C–60°C):EtOAc (v/v)); δ_H_ (400 MHz, CDCl_3_): 7.37 (2H, d, *J* = 8 Hz, benzyl 3-H), 7.24 (1H, dd, *J* = 8.5 & 7.5 Hz, benzyl 4-H), 4.92 (2H, s benzyl CH_2_); δ_C_ (100 MHz, CDCl_3_): 168.1 (thiadiazole 5-C), 138.1 (thiadiazole 2-C), 136.3 (benzyl 2-C), 131.5 (benzyl 1-C), 129.9 (benzyl 4-C), 128.6 (benzyl 3-C), 34.6 (benzyl CH_2_); m/z ES + Found MH^+^ 356.8462, C_9_H_5_BrCl_2_N_4_S_2_ requires MH^+^ 356.8512.

##### 3-(5-((2,6-dichlorobenzyl)thio)-1,3,4-thiadiazol-2-yl)aniline **KC156**

General procedure A was followed using 2-bromo-5-((2,6-dichlorobenzyl)thio)-1,3,4-thiadiazole (**7**) (100 mg, 0.28 mmol), 3-aminophenyl boronic acid (40 mg, 0.28 mmol), K_2_CO_3_ (155 mg, 1.12 mmol), Pd(PPh_3_)_4_ (35 mg, 0.03 mmol), 1,4-dioxane (2 mL) and water (2 mL) to afford a crude brown oil (165 mg). This was purified by ACC (0–60% EtOAc in Petroleum ether (40°C–60°C)) followed by HPLC (50–95% MeCN in H_2_O with a 0.1% formic acid additive) to afford a white solid (26 mg, 0.70 mmol, 25%). Rf 0.3 (7:3 Petroleum ether (40°C–60°C):EtOAc (v/v)); δ_H_ (400 MHz, D_6_-Acetone): 7.37 (2H, d, *J* = 7.5 Hz, benzyl 3-H), 7.27 (1H, dd, *J* = 9.0 & 7.0 Hz, benzyl 4-H), 7.16 (1H, ap. t, *J* = 2 Hz, anilinyl 2-H), 7.07 (1H, ap. t, *J* = 8.0 Hz, anilinyl 5-H), 7.00 (1H, ddd, *J* = 8.0, 2.0 & 1.0 Hz, 6-H), 4.79 (2H, s, benzyl CH_2_); δ_C_ (100 MHz, D_6_-Acetone): 170.0 (thiadiazolyl 2-C), 162.5 (thiadiazolyl 5-C), 149.4 (aniline 1-C), 135.8 (benzyl 2-C), 132.1 (benzyl 1-C), 130.5 (benzyl 4-C), 130.0 (aniline 5-C), 128.8 (benzyl 3-C), 118.2 (aniline 3-C), 117.1 (aniline 4-C), 116.0 (aniline 6-C), 112.5 (aniline 2-C), 34.5 (benzyl CH_2_); m/z ES + Found MH^+^ 367.9841, C15H11Cl2N3Ss requires MH^+^ 367.9844.

##### 4-(5-((2,6-dichlorobenzyl)thio)-1,3,4-thiadiazol-2-yl)aniline **KC146**

General procedure A was followed using 2-bromo-5-((2,6-dichlorobenzyl)thio)- 1,3,4-thiadiazole (**7**) (100 mg, 0.28 mmol), 4-aminophenyl boronic acid (39 mg, 0.28 mmol), K_2_CO_3_ (155 mg, 1.12 mmol), Pd(PPh_3_)_4_ (35 mg, 0.03 mmol), 1,4-dioxane (2 mL) and H_2_O (2 mL) to afford a crude brown oil (146 mg). This was purified by ACC (20–70% EtOAc in Petroleum ether (40°C–60°C)) to afford a yellow solid (22 mg, 0.06 mmol, 21%). Rf 0.30 (7:3 Petroleum ether (40°C–60°C):EtOAc (v/v)); δ_H_ (400 MHz, CDCl_3_): 7.63 (1H, d, *J* = 8.5 Hz, phenyl 2-H), 7.26 (2H, d, *J* = 8 Hz, benzyl 3-H), 7.12 (1H, dd, *J* = 8.5 & 7.5 Hz, benzyl 4-H), 6.64 (d, *J* = 8.5 Hz, phenyl 3-H), 4.84 (2H, s, benzyl CH_2_); δ_C_ (100 MHz, CDCl_3_): 169.8 (thiadiazole 2-C), 167.8 (thiadiazole 5-C), 149.3 (phenyl 4-C), 136.3 (benzyl 2-C), 132.2 (benzyl 2-C), 129.6 (benzyl 4-C), 129.4 (phenyl 2-C), 128.5 (benzyl 3-C), 120.1 (phenyl 4-C), 114.9 (phenyl 3-C), 34.8 (benzyl CH_2_); m/z ES + Found MH^+^ 367.9893, C_15_H_11_Cl_2_N_3_S_2_ requires MH^+^ 367.9850.

##### 3-(5-((2,6-Dichlorobenzyl)thio)-1,3,4-thiadiazol-2- yl)benzonitrile **KC183**

General procedure A was followed using 2-bromo-5-((2,6-dichlorobenzyl)thio)- 1,3,4-thiadiazole (**7**) (100 mg, 0.28 mmol), (3-cyanophenyl)boronic acid (37 mg, 0.28 mmol), K_2_CO_3_ (155 mg, 1.12 mmol), Pd(PPh_3_)_4_ (35 mg, 0.03 mmol), 1,4-dioxane (2 mL) and H_2_O (2 mL) to afford a crude brown solid (390 mg). This was purified by ACC (0–60% ethyl acetate in petroleum ether (40°C–60°C)) to afford a yellow solid (25 mg, 0.05 mmol, 18%). δ_H_ (400 MHz, CDCl_3_): 8.12 (1H, s, benzonitrilyl 2-H), 8.07 (1H, d, *J* = 8.0 Hz, benzonitrilyl 4-H), 7.70 (1H, d, *J* = 8.0 Hz, benzonitrilyl 6-H), 7.55 (1H, ap. *t*, J = 8.0 Hz, benzonitrilyl 5-H), 7.29 (2H, d, *J* = 8.0 Hz, benzyl 3-H), 7.15 (1H, t, *J* = 8.0 Hz, benzyl 4-H), 4.92 (2H, s, benzyl CH_2_); δ_C_ (100 MHz, CDCl_3_): 166.4 (thiadiazolyl 2/5-C), 165.6 (thiadiazolyl 2/5-C), 136.3 (benzyl 2-C), 134.1 (benzonitrilyl 6-C), 131.6 (benzonitrilyl 4-C), 131.6 (benzyl 1-C), 131.2 (benzonitrilyl 1-C), 131.1 (benzonitrilyl 2-C), 130.2 (benzonitrilyl 5-C), 129.9 (benzyl 4-C), 128.6 (benzyl 3-C), 117.7 (nitrile), 113.8 (benzonitrilyl 3-C), 34.7 (benzyl CH_2_); m/z ES + Found MH^+^ 377.9685, C_16_H_9_Cl_2_N_3_S_2_ requires MH^+^ 377.9688.

##### Ethyl 2-([(2,6-dichlorophenyl)methyl]sulfanyl)-1,3,4-thiadiazole-5-carboxylate (**8**)

To a solution of 2,6-dichlorobenzyl thiol (**5**) (502 mg, 2.60 mmol) and ethyl 5-bromo-1,3,4-thiadiazole-2-carboxylate (570 mg, 2.41 mmol) in an. DMF (8 mL) under N_2_ was added Et_3_N (0.40 mL, 2.89 mmol) and the reaction heated to 90°C for 21h. The reaction was then cooled to RT and diluted with H_2_O (100 mL). The aqueous solution was extracted with EtOAc (2 × 70 mL) and then combined organic phases washed with brine (70 mL) and 10% LiCl solution (70 mL), dried (Na_2_SO_4_) and concentrated *in vacuo*. ACC (0–20% EtOAc in petroleum ether (40°C–60°C)) to give the title compound as a white solid (670 mg, 1.92 mmol, 80%). Rf 0.66 (8:2 Petroleum ether (40°C–60°C):EtOAc (v/v)); δ_H_ (400 MHz, CDCl_3_): 7.28 (2H, d, *J* = 8.1 Hz, benzyl H-3/5), 7.17-7.13 (1H, m, benzyl H-4), 4.94 (2H, s, benzylic CH_2_), 4.43 (2H, q, *J* = 7.1 Hz, C*H*_2_CH_3_), 1.38 (3H, t, *J* = 7.12 Hz, CH_2_C*H*_3_); δ_C_ (100 MHz, CDCl_3_): 170.4 (thiadiazole C-2), 160.2 (C=O), 158.5 (thiadiazole C-5), 136.3 (benzyl C-2/6), 131.3 (benzyl C-1), 130.0 (benzyl C-4), 128.6 (benzyl C-3/5), 63.3 (*C*H_2_CH_3_), 34.5 (benzylic CH_2_), 14.2 (CH_2_*C*H_3_); m/z ES + Found MH^+^ 348.9631, C_12_H_11_Cl_2_N_2_O_2_S_2_ requires MH^+^ 348.9633.

##### (2-([(2,6-Dichlorophenyl)methyl]sulfanyl)-1,3,4-thiadiazol-5-yl)(piperidin-1-yl)methanone **CHR-1741-077**

To a solution of ethyl 2-([(2,6-dichlorophenyl)methyl]sulfanyl)-1,3,4-thiadiazole-5-carboxylate (**8**) (55 mg, 0.16 mmol) in EtOH (4 mL) under N_2_ was added piperidine (0.08 mL, 0.79 mmol) and the reaction heated to reflux. After 3h the reaction was cooled to RT and volatile solvents removed *in vacuo*. The crude mixture was dissolved in H_2_O (30 mL) and extracted with EtOAc (3 × 40 mL), combined organic phases were washed with brine (30 mL), dried (Na_2_SO_4_) and concentrated *in vacuo*. ACC (0–30% EtOAc in petroleum ether (40°C–60°C)) to give the title compound as a white solid (24 mg, 0.062 mmol, 39%). Rf 0.48 (8:2 Petroleum ether (40°C–60°C):EtOAc (v/v)); δ_H_ (400 MHz, CDCl_3_): 7.28 (2H, d, *J* = 8.04 Hz, benzyl H-3/5), 7.17-7.13 (1H, m, benzyl H-4), 4.90 (2H, s, benzylic CH_2_), 4.15-4-13 (2H, m, piperidine), 3.67-3.65 (2H, m, piperidine), 1.70-1.65 (6H, m, piperidine); δ_C_ (100 MHz, CDCl_3_): 169.0 (thiadiazole C-2), 167.0 (thiadiazole C-5), 156.8 (C=O), 136.3 (benzyl C-2/6), 131.3 (benzyl C-1), 129.9 (benzyl C-4), 128.6 (benzyl C-3/5), 47.8 (piperidine *C*HN), 44.8 (piperidine *C*HN), 34.2 (benzylic CH_2_), 26.7 (piperidine), 25.8 (piperidine), 24.5 (piperidine); m/z ES + Found MNa^+^ 409.9930, C_15_H_15_Cl_2_N_3_NaOS_2_ requires MNa^+^ 409.9925.

##### 2-([(2,6-Dichlorophenyl)methyl]sulfanyl)-1,3,4-thiadiazol-5-amine (**9**)

To a solution of 2-amino-5-bromo-1,3,4-thiadiazole (557 mg, 3.09 mmol) and 2,6-dichlorobenzyl thiol (**5**) (717 mg, 3.71 mmol) in an. DMF (15 mL) under N_2_ was added Et_3_N (0.52 mmol, 3.71 mmol) and the reaction heated to 90°C. After heating for 20h reaction was cooled to RT and diluted with H_2_O (100 mL). The resulting precipitate was collected by filtration. ACC (30–70% EtOAc in petroleum ether (40°C–60°C)) to give the title compound as a white solid (665 mg, 2.28 mmol, 74%). Rf 0.28 (1:1 Petroleum ether (40°C–60°C):EtOAc (v/v)); δ_H_ (400 MHz, d_6_-DMSO): 7.51 (2H, d, *J* = 7.8 Hz, benzyl H-3/5), 7.42-7.35 (3H, m, benzyl H-4 and NH_2_), 4.46 (2H, s, benzylic CH_2_); δ_C_ (100 MHz, d_6_-DMSO): 171.8 (thiadiazole C-5), 147.6 (thiadiazole C-5), 135.4 (benzyl C-2/6), 133.2 (benzyl C-1), 130.8 (benzyl C-4), 129.2 (benzyl C-3/5), 36.0 (benzylic CH_2_); m/z ES + Found MH^+^ 291.9529, C_9_H_8_Cl_2_N_3_S_2_ requires MH^+^ 291.9531.

##### *N*-(5-([(2,6-Dichlorophenyl)methyl]sulfanyl)-1,3,4-thiadiazol-2-yl)-1,2-oxazole-5-carboxamide **CHR-1871-005**

To a suspension of 2-([(2,6-dichlorophenyl)methyl]sulfanyl)-1,3,4-thiadiazol-5-amine (**9**) (59 mg, 0.20 mmol) in anhydrous THF (4 mL) under N_2_ was added DMAP (1 mg, 0.01 mmol) Et_3_N (0.03 mL, 0.22 mmol) followed by isoxazole-5-carbonyl chloride (0.03 mL, 0.30 mmol). The reaction was stirred at room temperature for 20 h, then diluted with water (30 mL). The aqueous solution was extracted with EtOAc (3 × 20 mL), the combined organic phases were washed with brine (20 mL), dried (Na_2_SO_4_) and concentrated *in vacuo*. ACC (50–100% EtOAc in Petroleum ether (40°C–60°C)) to afford a white residue (52 mg, 0.13 mmol, 67%). Rf 0.56 (3:7 Petroleum ether (40°C–60°C):EtOAc (v/v)); δ_H_ (400 MHz, d_6_-DMSO): 13.87 (1H, br. s, NH), 8.88 (1H, s, isoxazole H-3), 7.55-7.53 (3H, m, isoxazole H-4 and benzyl H-3/5), 7.43-7.39 (1H, m, benzyl H-4), 4.70 (2H, s, benzylic CH_2_); δ_C_ (125 MHz, d_6_-DMSO): 160.9 (thiadiazole C-5), 157.7 (thiadiazole C-2 or isoxazole C-5), 155.2 (C=O), 152.5 (isoxazole C-3). 135.6 (benzyl C-2/6), 132.5 (benzyl C-1), 131.1 (benzyl C-4), 129.3 (benzyl C-3/5), 108.8 (isoxazole C-4), 35.1 (benzylic CH_2_); m/z ES + Found MH^+^ 386. 9534, C_13_H_8_Cl_2_N_4_O_2_S_2_ requires 386.9538 MH^+^.

### Quantification and statistical analysis

Averaged data are shown as mean ± standard error of the mean (s.e.m.) or standard deviation (s.d.), as specified in the figure legends. Data were produced in pairs and analyzed statistically with Student’s t test or in groups and analyzed by one-way ANOVA using OriginR2018 software (OriginLab Corporation, USA). Statistically significant difference is indicated by ∗*p* < 0.05. n.s., indicates not significantly different. “n” is used to denote the number of independent data points from independent patch-clamp or Ca^2+^ recordings. Representative traces contained “N” technical replicates from a single experiment. For patch-clamp studies, data were analyzed using pClamp version 10.6 (Molecular Devices, USA) and Origin^R^2018 software packages.

## Supplemental information

Document S1. Figures S1–S9 and Tables S1 and S2

Data S1. Original data for laboratory results
