# Supplementary material for: PIEZO1 mechanical insensitivity in generalized lymphatic dysplasia with the potential for pharmacological rescue
Source: iScience. 2025 Jul 15;28(8):113110. doi: 10.1016/j.isci.2025.113110 (PMC12337661; doi:10.1016/j.isci.2025.113110)
Supplement: Document S1. Figures S1–S9 and Tables S1 and S2 [file mmc1.pdf]

iScience, Volume *28*

Supplemental information

PIEZO1 mechanical insensitivity in generalized lymphatic dysplasia with the potential for pharmacological rescue

Melanie J. Ludlow, Oleksandr V. Povstyan, Deborah M. Linley, Silvia Martin-Almedina, Charlotte Revill, Kevin Cuthbertson, Katie A. Smith, Emily Fay, Elisavet Fotiou, Andrew Bush, Claire Hogg, Tobias Linden, Natalie B. Tan, Susan M. White, Juan C. Del Rey Jimenez, Ege Sackey, Esther Dempsey, Sahar Mansour, Gregory Parsonage, Antreas C. Kalli, Richard Foster, Pia Ostergaard, and David J. Beech

ID	Variant*	Genomic coordinates (GRCh38)	Exon	PIEZO1 region	REVEL	Alpha-Missense	CADD v1.7	Splice AI	GnomAD v4	Clinvar	ACMG Pathogenicity criteria	Classification
GLD 7	c.5933G>A G1978D	chr16-88720401 C>T	41	THU9	0.86	0.989	27.0	0	1 Het in 775116 controls	Absent	PM2, PP3, PS3_Moderate, PP4	LP (6 points)
GLD 7	c.7004G>A R2335Q	chr16-88716406 C>T	48	Cap	0.32	0.472	24.5	0.01	15 Het in 774574 controls	Absent	PM2, BP4, PS3_Moderate, PM3, PP4	LP (6 points)
GLD 8 GLD 9	c.6809T>C I2270T	chr16-88716676 A>G	47	Cap	0.65	0.896	23.9	0	10 Het in 774580 controls	VUS	PM2, PP3, PM3, PS3_Moderate, PP1_Moderate, PP4	P (10 points)
GLD 9	c.2486A>T E829V	chr16-88733589 T>A	18	THU5	0.45	0.932	35	0.68	1 Het in 770080 controls	Absent	PM2, PS3_ Moderate, PM3, PP3, PP4	LP (8 points)

SI Table S1: Interpretation of the pathogenicity of *PIEZO1* variants identified in this study, complementing the information and discussion in the main paper

Annotation, *in silico* predictions, population allele frequencies and pathogenicity are summarized. Association for Clinical Genomic Science (ACMG) pathogenicity scores include the functional evidence collected in this study under the PS3 criterion (see also Table 2 of the main paper).

*Variants are annotated to the NM_001142864.4 reference transcript and displayed as nucleotide and amino acid changes. GLD, Generalised Lymphatic Dysplasia; THU, transmembrane helical unit; Cap, C-terminal Extracellular Domain (CED); Het, Heterozygous; VUS, Variant of Uncertain Significance; P; Pathogenic, LP, Likely Pathogenic.

REVEL [S1] is a metapredictor that combines 13 prediction tools with scores ranging from 0 to 1. Thresholds are: benign (<0.4), pathogenic (≥0.7).

AlphaMissense [S2] is a deep learning model that classifies missense variants as likely pathogenic (>0.564), likely benign (<0.34), or ambiguous (0.34–0.564).

CADD v1.7 [S3] provides a PHRED-like score ranging from 1 to 99, with higher values indicating more deleterious variants.

SpliceAI [S4] predicts the probability (0 to 1) that a variant affects splicing with a threshold for mis-splicing at >0.2.

GnomAD v4 [S5]: Allele frequency in controls was extracted from the Genome Aggregation Database (gnomAD) v4.1, which comprises sequencing data from 807,162 controls.

Clinvar [S6]: public archive of relationships among sequence variation and human phenotype.

ACMG Pathogenicity Criteria: Variant classifications used in this study are based on the criteria described in the ACMG variant interpretation guidelines [S7], such as PM3 (e.g., for recessive disorders, detected in trans with a pathogenic variant), and follows the ACGS 2024 [S8] specifications of these guidelines. Variants were categorised in this study as Pathogenic (P), ≥10 points or Likely Pathogenic (LP), 6-9 points.

Human PIEZO1 (hPIEZO1)		
E829V	Forward (5' - 3')	GGTGGCCCTGAAGGTGGTGTCGGTGATGAAC
	Reverse (5' - 3')	GTTCATCACCGACACCACCTTCAGGGCCACC
G1978D	Forward (5' - 3')	CATCATCATCATTTTTGACTTCTGGGCCTTTGGG
	Reverse (5' - 3')	CCCAAAGGCCCAGAAGTCAAAAATGATGATGATG
I2270T	Forward (5' - 3')	GTCACGGCGCAGACTGAGGGCAGCTC
	Reverse (5' - 3')	GAGCTGCCCTCAGTCTGCGCCGTGAC
R2335Q	Forward (5' - 3')	CAACAGCACTGCACAGCGGCAGCTGGCCAG
	Reverse (5' - 3')	CTGGCCAGCTGCCGCTGTGCAGTGCTGTTG

Mouse PIEZO1 (mPIEZO1)		
E824V	Forward (5' - 3')	GGTGGCCCTGAAAGTAGTGTCTGTGATGAAC
	Reverse (5' - 3')	GTTCATCACAGACACTACTTTCAGGGCCACC
G1994D	Forward (5' - 3')	CATCATCATCATCATCTTTGATTTTTGGGCTTTTGGGAAGC
	Reverse (5' - 3')	GCTTCCCAAAAGCCCAAAAATCAAAGATGATGATGATGATG
I2286T	Forward (5' - 3')	CGTCACTGCACAGACCGAGGGCAGCTCGG
	Reverse (5' - 3')	CCGAGCTGCCCTCGGTCTGTGCAGTGACG
R2351Q	Forward (5' - 3')	CAACAGTACGGCACAAAGGCAGCTGGCCC
	Reverse (5' - 3')	GGGCCAGCTGCCTTTGTGCCGTACTGTTG

SI Table S2: Primer sequences used to generate the PIEZO1 variants as indicated in the STAR * Methods.

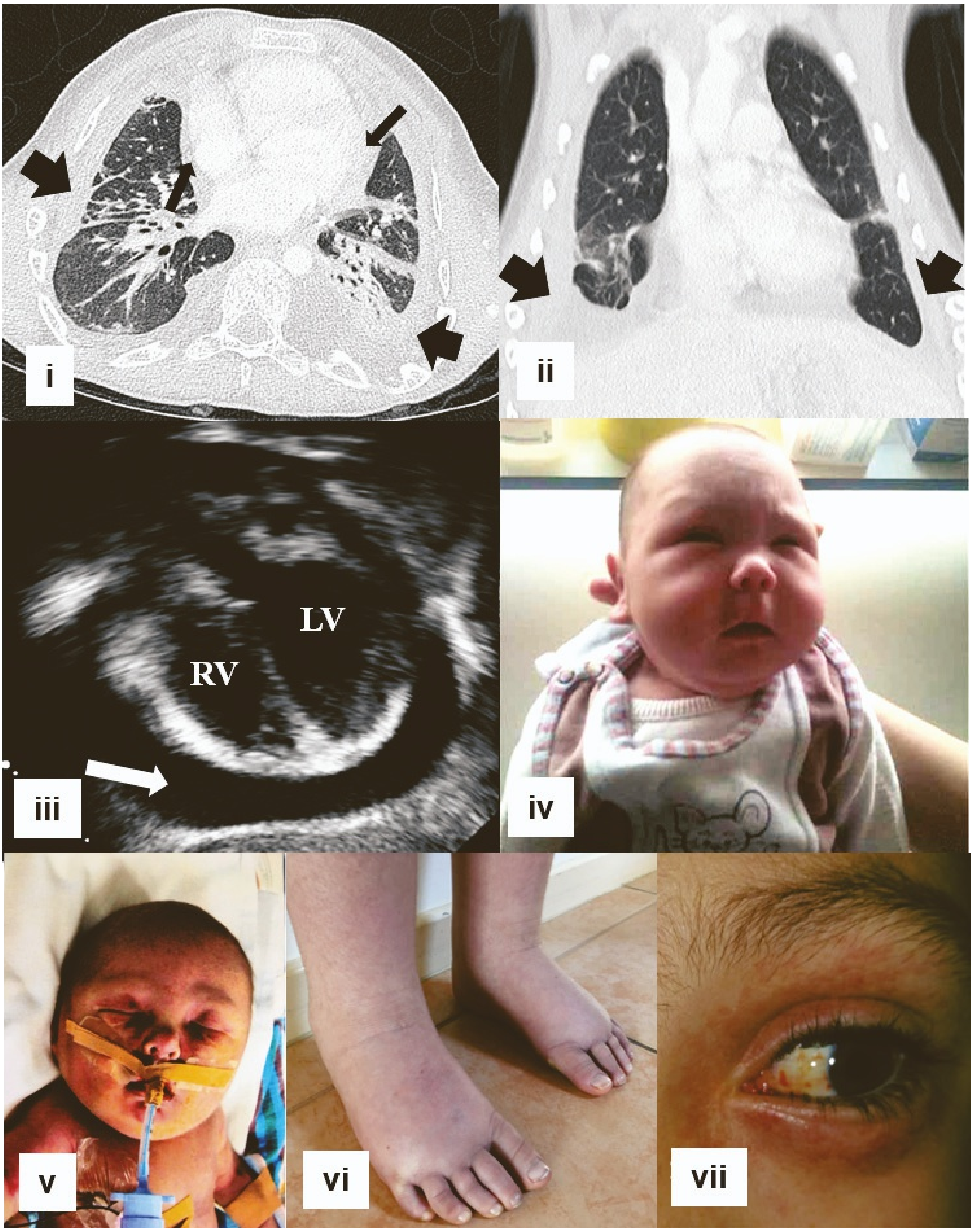

SI Figure S1: Clinical images of the GLD families complementing the patient information in the main paper

(**i**) – (**iii**) Proband (GLD08 II.1). In (**i**) and (**ii**), computerised tomography (CT) of thorax demonstrating bilateral chylothorax (thick arrows) and a pericardial effusion (thin arrows) and in (**iii**) a large pericardial effusion (white arrow) requiring percutaneous drainage twice for recurrence. RV, right ventricle. LV, left ventricle. (**iv**) Proband (GLD07 II.1) showing facial edema in infancy. (**v**) – (**vii**) Proband (GLD09 II.1). In (**v**) showing fetal hydrops at birth, (**vi**) bilateral lower limb lymphedema in early childhood and (**vii**) bilateral periorbital and conjunctival vascular changes with small punctate haemorrhages.

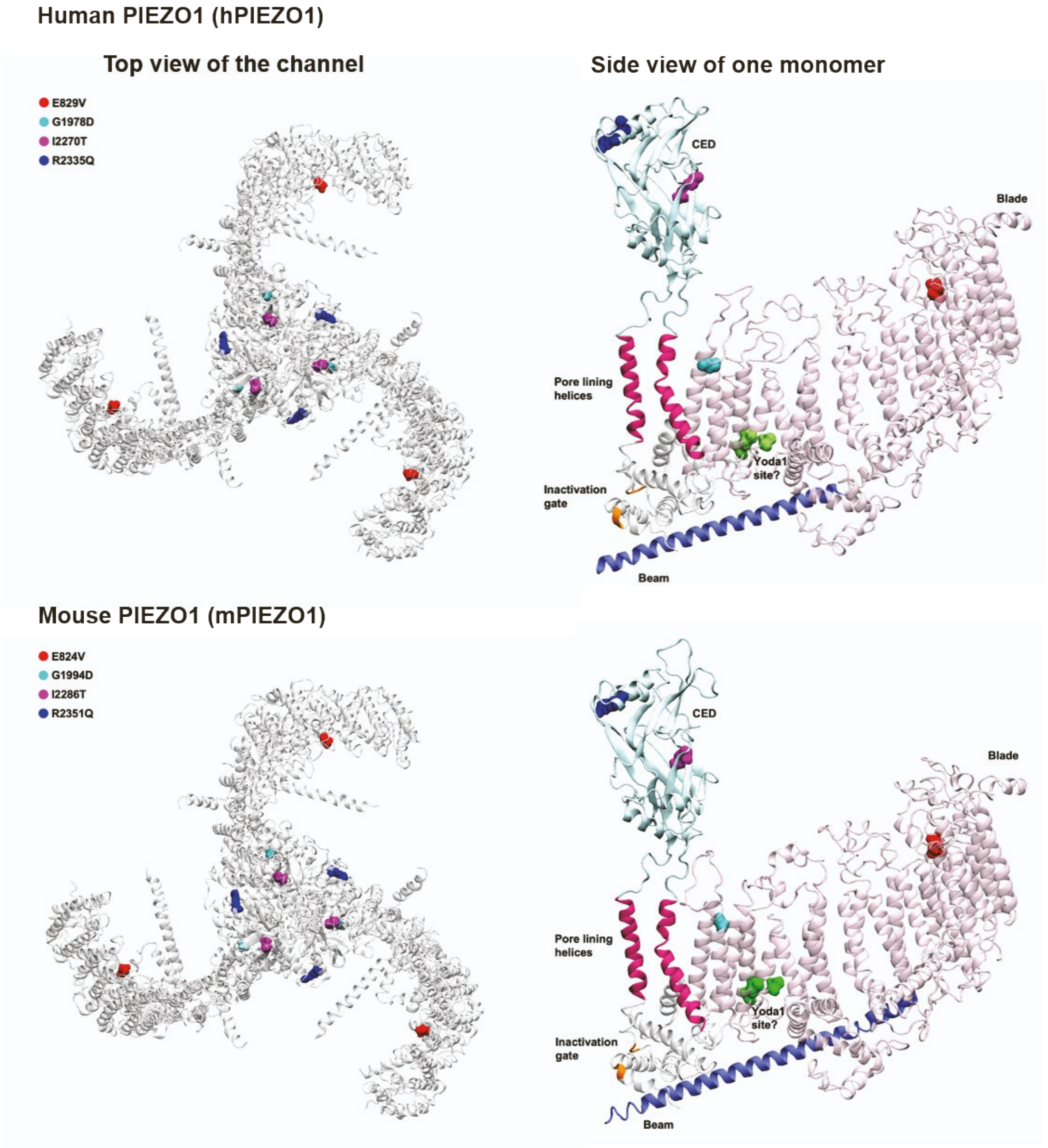

SI Figure S2: Molecular models of hPIEZO1 and mPIEZO1 channels as described in the main paper

The channel is shown using cartoon representation with mutated residues displayed as surface representation according to the colour-code indicated.

Top: human PIEZO1 (hPIEZO1) channel.

Bottom: mouse PIEZO1 (mPIEZO1) channel.

On the left in each case is the trimeric channel in helicopter view.

On the right in each case is one PIEZO1 monomer shown using a side view.

Key features of the channels are indicated: C-terminal extracellular domain (CED, pale blue); pore lining helices (pink); inactivation gate (orange); beam (blue); blade (pale pink); and potential Yoda1 interaction amino acid residues (green) suggested by mutagenesis studies.

```
                     G1978               R2335               E829                I2270
HUMAN        VDFIIIIFGFWAFGKHS   ALAPNSTARRQLASLLE   YTVWVALKEVSVMNLLL   PEDIVTAQIEGSSGALW
CHIMP        VDFIIIIFGFWAFGKHS   ALAPNSTARQQLASLLE   YTVWVALKEVSVMNLLL   PEDIVTAQIEGSSGALW
MONKEY       VDFIIIIFGFWAFGKHS   ALAPNSTERRQLASLLE   YTVWVALKEVSVMNLLL   PEDIVTAQIEGSSGALW
MOUSE        VDIIIIIFGFWAFGKHS   ELAPNSTARRQLAQLLE   YTVWVALKEVSVMNLLL   PEDIVTAQIEGSSGALW
RAT          VDIVVIIFGFWAFGKHS   ELAPNSTARRQLAQLLE   YTVWVALKEVSVMNLLL   PEDIVTAQIEGSSGALW
HAMSTER      VDFIIIVFGFWAFGKHS   ELAPNSTARRQLAQLLE   YTVWVALKEVSVMNLLL   PEDIVTAQIEGSSGALW
DOG          VDFIIIIFGFWAFGKHS   DWAPNSTERRQLASLLE   YTVWVALKEVSVMNLLL   PEDIVTAQIEGSSGALW
SHEEP        IDFIIIIFGFWAFGKHS   DLAPNSTARRQLASLLE   YTVWVALKEVSVLNFLL   PEDIVTVHIEGSSGALW
GOAT         IDFIIIIFGFWAFGKHS   DLAPNSTARRQLASLLE   YTVWVALKEVSVLNFLL   PEDIVTVHIEGSSGALW
COW          IDFIIIIFGFWAFGKHS   DLAPNSTARRQLASLLE   YTVWVALKEVSVLNFLL   PEDIVTVHIEGSSGALW
KOALA        IDFIIIVFGFWAFGKHS   DLTPNSTERRQLANLLE   YTVWVALKEVSVMNFLL   YEDIVTAQIEGSSGALW
OPOSSUM      VDFIIIVFGFWAFGKHS   DLTPNSTERRQLASLLE   YTVWVALKEVSVMNFLL   YEDIVTAQIEGSSGALW
GUINEAFOWL   VDFIIIIFGFWAFGKHS   DLQPGAPERMELAQLLQ   YTVWVALQEVSLMNFLL   YEDIVTARIEGSSGSLW
ZEBRAFISH    VDFIIIIFGFWAFGKHS   SLAPKNPVRADLASLLL   FVVWVALLEPSAMNLVL   YEDIVTANIEGSSGSVW
XENOPUS      VDFVIIVFGFWAFGKHS   DLEPGSQERMELADLLQ   YSVWVALQEVSVMNFLF   YEDIVTARIEGSSGSVW
             :*:::*:**********   *    * :**.**      : ***** * * :*:::    *****..******::*

Hs PIEZO1    VDFIIIIFGFWAFGKHS   ALAPNSTARRQLASLLE   YTVWVALKEVSVMNLLL   PEDIVTAQIEGSSGALW
Hs PIEZO2    VDFIIIVFGFWAFGKHS   PL--KNITRKNIAKMIA   YIIWVSVKEVSLFNYVF   KEDITVAELEGNSNSLW
             ******:**********    *  :. :*:::*.::   * :**::****::* ::    ***..*::**.*.:**
```

SI Figure S3: Amino acid sequence conservation at the variant sites as described in the main paper

Upper panel: Single-letter amino acid code for sections of PIEZO1 amino acid sequences at and around the sites of the 4 GLD-associated variants after amino acid identity alignments of sequences of the 15 species indicated on the left. The wild-type residues are indicated across the top for the sites of the 4 variants in human PIEZO1 (hPIEZO1) and shown in red across the 15 species. Asterisks at the bottom indicate perfect sequence conservation across the species.

Lower panel: Alignment results for human (Homo sapiens, Hs) PIEZO1 and PIEZO2.

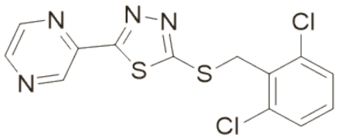

Yoda1

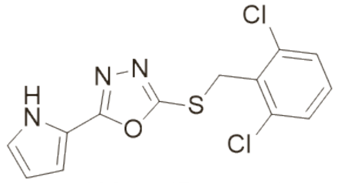

KC41 (Dooku1)

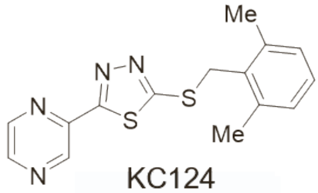

KC124

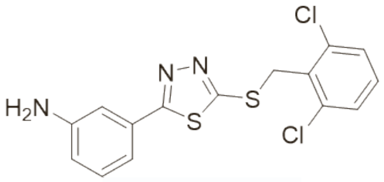

KC156

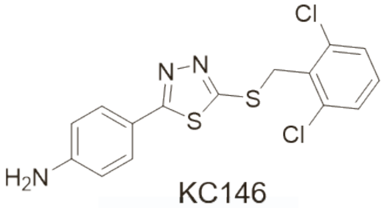

KC146

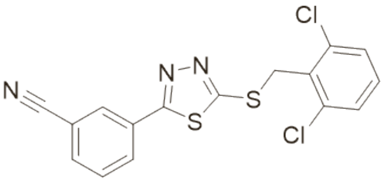

KC183

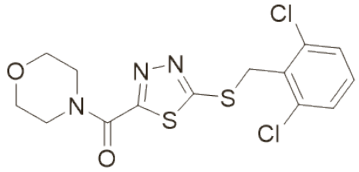

CHR-1741-077

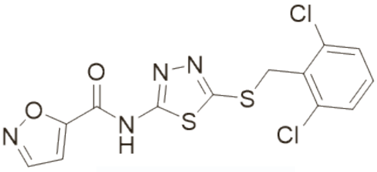

CHR-1871-005

SI Figure S4: Chemical structures of 6 of the novel Yoda1 analogues generated in this study and described in the main paper. The structure of Yoda2b (CHR-1871-032) is provided in Figure 3j. Five compounds retained the thiadiazole central ring and the 2,6-dichlorobenzylthiol ether moiety of Yoda1 but replace the pyrazinyl group with either a substituted phenyl ring (compounds KC156, KC146 and KC183) or a substituted amide (CHR-1741-077 and CHR-1871-005). KC124 retains the thiadiazole central ring and pyrazinyl ring of Yoda1 but replaces the 2,6-dichlorobenzylthiol ether moiety with a 2,6-dimethylbenzylthiol ether moiety.

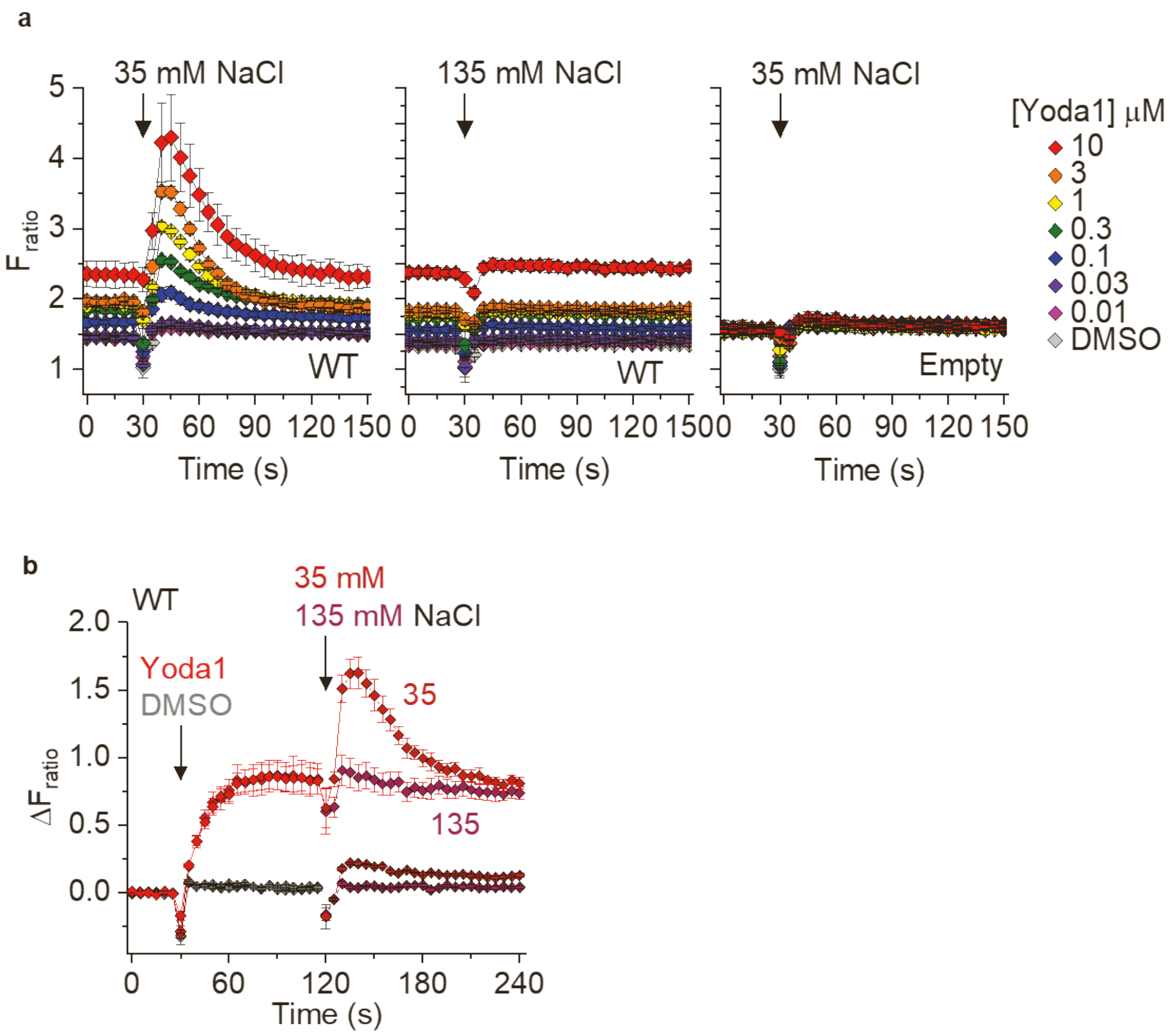

SI Figure S5: Hypo-osmolality test in the Ca^{2+} assay, referred to in the main paper

(**a**) Typical 96-well plate mean ± s.e.m. data for changes in intracellular Ca^{2+} evoked by hypotonic (35 mM NaCl) or normotonic (135 mM NaCl) medium for WT- or Empty vector-transfected cells in increasing concentrations of Yoda1 as indicated and colour-coded on the right (representative of n = 3-6 independent experiments).

(**b**) For WT channels, mean ± s.e.m. responses to 0.1 μM Yoda1 or vehicle control (DMSO) and subsequent challenge with normotonic (135 mM NaCl) or hypotonic (35 mM NaCl) solution (in equal volume) according to the colour code indicated (N = 4-5 wells each).

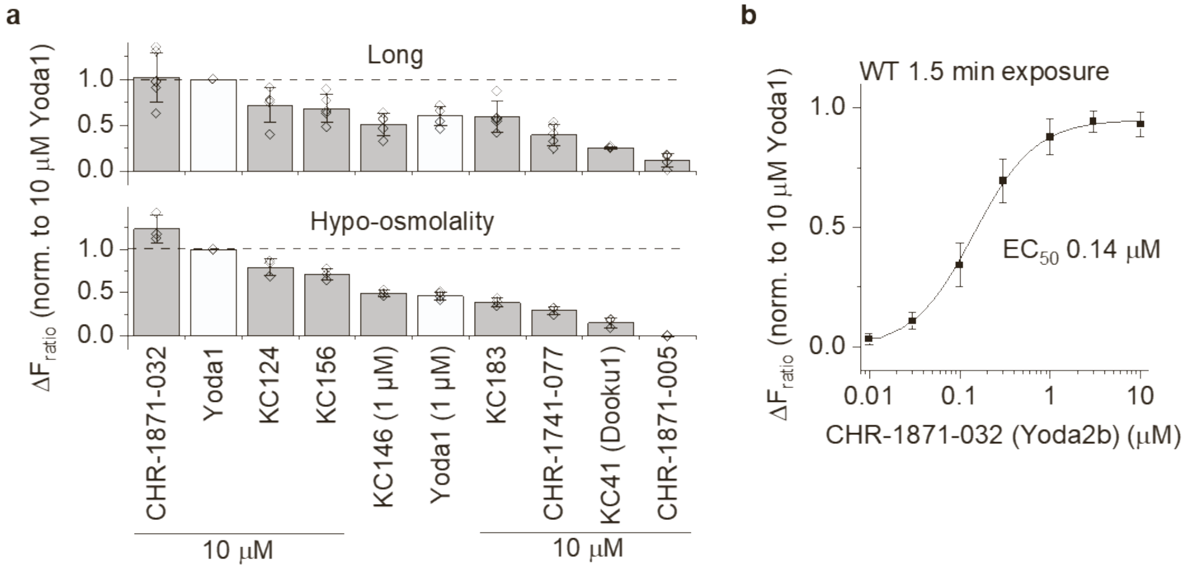

SI Figure S6: Effects of novel Yoda1 analogues tested against variant channels referred to in the main paper

(**a**) Long substance exposure and hypo-osmolality protocol mean ± s.d. Ca^{2+} data for the indicated Yoda1 analogues tested against the indicated variants (n = 3-8 independent experiments). All analogues were tested at 10 μM except for KC146, which was tested at 1 μM because it caused a fluorescence artefact at 10 μM. Chemical structures of the analogues are in SI Figure S4. Yoda2b is CHR-1871-032. The hypo-osmolality protocol is described in SI Figure S5.

(**b**) Mean ± s.e.m. concentration-response data for CHR-1871-032 (Yoda2b) against WT mPIEZO1 using 1.5 min exposure (n = 3 independent experiments).

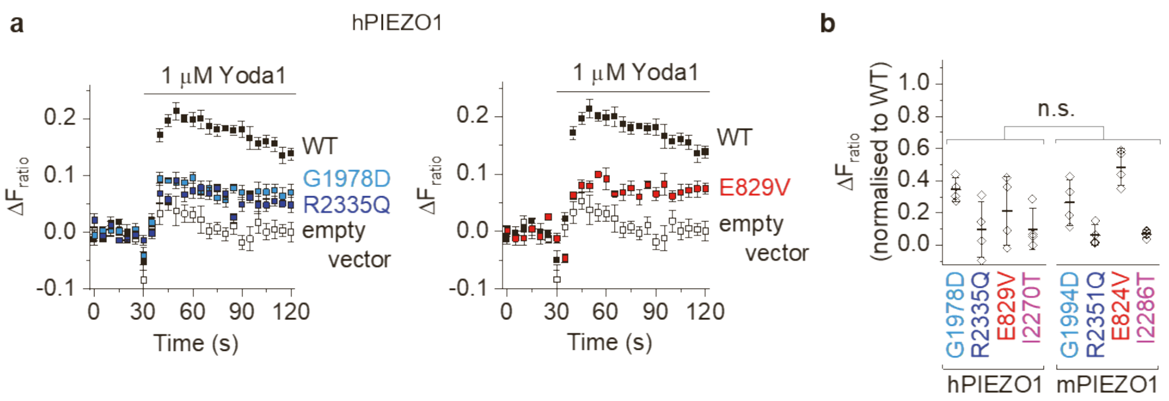

SI Figure S7: Effects of variants on responses to 1 μM Yoda1

Further data of the type shown in Figure 2c.

(**a**) Increase in intracellular Ca^{2+} concentration indicated by increase in the fura-2 fluorescence (F) ratio above baseline (ΔF_{ratio}) in T-REx-293 cells transiently transfected with wild-type (WT) hPIEZO1, G1978D hPIEZO1, R2335Q hPIEZO1, E829V hPIEZO1or empty vector (vector control). Cells were stimulated with 1 μM Yoda1. Example data are shown for a single representative 96-well plate experiments (mean ± s.e.m., N = 4-5 wells each).

(**b**) Summary data for experiments of the type shown in (**a**) and for all 4 variants recapitulated in mPIEZO1. The Ca^{2+} response was measured between 30 and 60 seconds (s) after Yoda1 application (n = 4 independent experiments each). The I2270T hPIEZO1 data are repeated from Figure 2c. Data are mean ± s.d. normalised to the respective WT channel data and subtracted for the amplitude in the response in the empty vector group; n.s. indicates no statistically significant difference between the responses for hPIEZO1 and mPIEZO1 variants (one-way ANOVA).

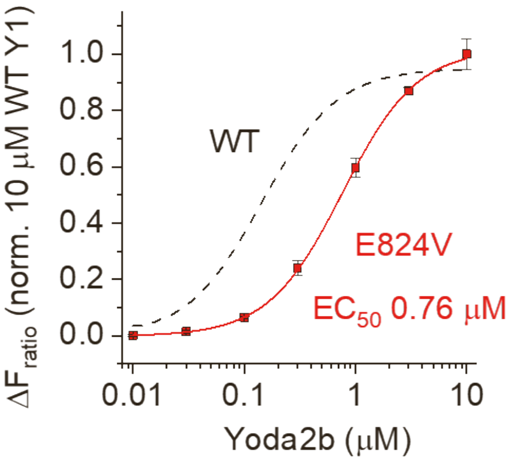

SI Figure S8: Pharmacological activation of E824V mPIEZO1 referred to in the main paper

Mean ± s.e.m. data for the intracellular Ca^{2+} concentration indicated by the increase in the fura-2 fluorescence (F) ratio above baseline (ΔF_{ratio}) in T-REx-293 cells stably transfected with E824V mPIEZO1 (red-coloured data, n = 3 independent experiments). The data are normalized to the WT Yoda1 maximum response and fitted with the Hill equation, indicating an EC_{50} of 0.76 μM for E824V. For comparison, the fitted Hill equation for the WT channel data is superimposed as a dashed curve (from SI Figure S6b).

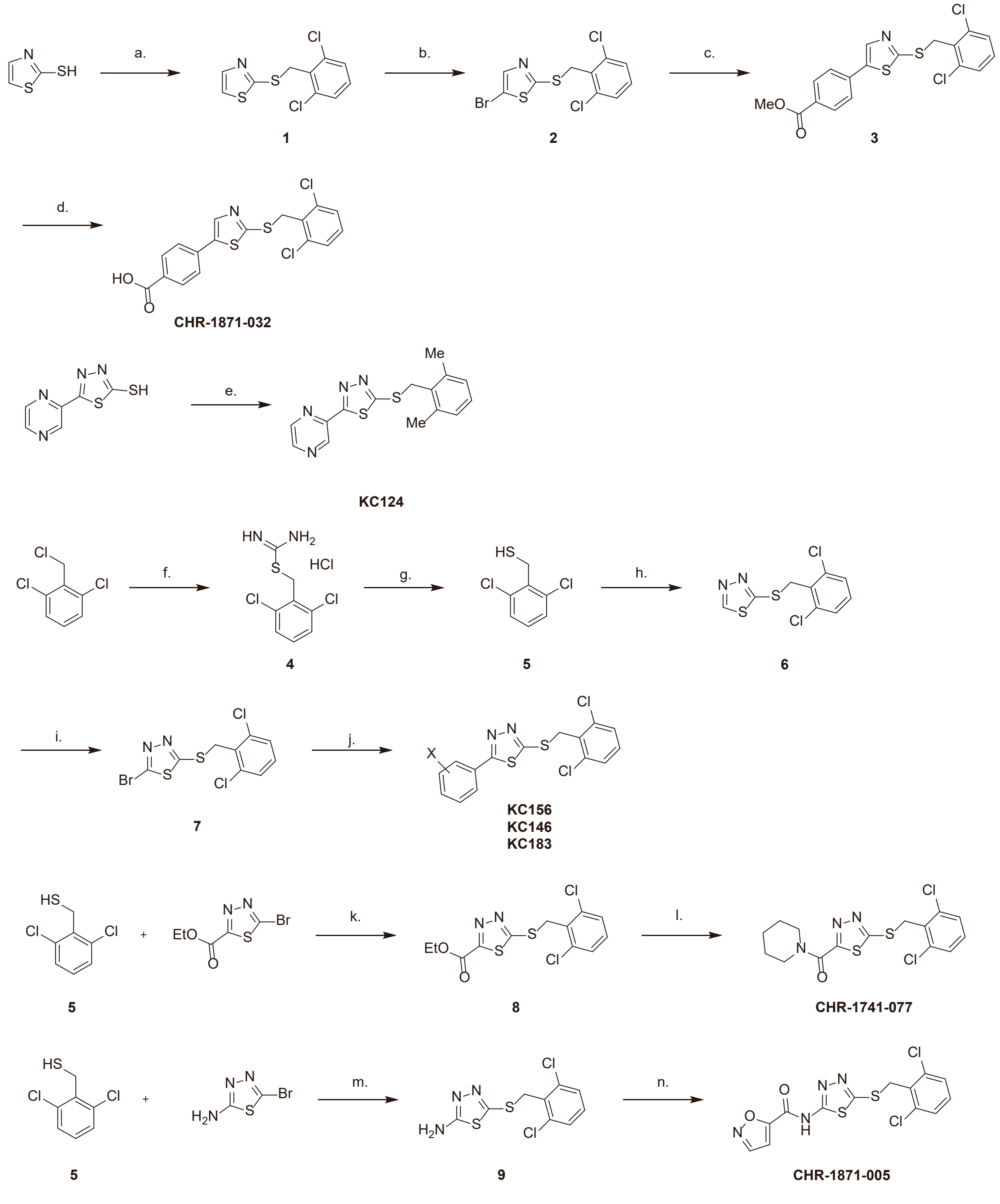

SI Figure S9: Scheme 1 for the STAR * Methods.

References

[S1] Ioannidis NM, Rothstein JH, Pejaver V, Middha S, McDonnell SK, Baheti S, et al. REVEL: An Ensemble Method for Predicting the Pathogenicity of Rare Missense Variants. *Am. J. Human Genetics*. 2016 99(4):877–85.

[S2] Cheng J, Novati G, Pan J, Bycroft C, Žemgulytė A, Applebaum T, et al. Accurate proteome-wide missense variant effect prediction with AlphaMissense. *Science*. 2023 381(6664):eadg7492.

[S3] Kircher M, Witten DM, Jain P, O'Roak BJ, Cooper GM, Shendure J. A general framework for estimating the relative pathogenicity of human genetic variants. *Nat Genet*. 2014 46(3):310–5.

[S4] Jaganathan K, Kyriazopoulou Panagiotopoulou S, McRae JF, Darbandi SF, Knowles D, Li YI, et al. Predicting Splicing from Primary Sequence with Deep Learning. *Cell*. 2019 176(3):535-548.e24.

[S5] Chen S, Francioli LC, Goodrich JK, et al. A genomic mutational constraint map using variation in 76,156 human genomes [published correction appears in Nature. 2024 Feb;626(7997):E1]. Nature. 2024;625(7993):92-100.

[S6] Landrum MJ, Lee JM, Riley GR, Jang W, Rubinstein WS, Church DM, et al. ClinVar: public archive of relationships among sequence variation and human phenotype. Nucleic Acids Res. 2014 Jan;42(Database issue). Available from: https://www.ncbi.nlm.nih.gov/clinvar/.

[S7] Richards S, Aziz N, Bale S, Bick D, Das S, Gastier-Foster J, et al. Standards and guidelines for the interpretation of sequence variants: a joint consensus recommendation of the American College of Medical Genetics and Genomics and the Association for Molecular Pathology. *Genet Med*. 2015 17(5):405–24.

[S8] Durkie M, Cassidy EJ, Berry I, Owens M, Turnbull C, Scott R, et al. ACGS Best Practice Guidelines for Variant Classification in Rare Disease 2024. Available from: https://www.acgs.uk.com/media/12533/uk-practice-guidelines-for-variant-classification-v12-2024.pdf
